# TNFRSF receptor-specific antibody fusion proteins with targeting controlled FcγR-independent agonistic activity

**DOI:** 10.1038/s41419-019-1456-x

**Published:** 2019-03-04

**Authors:** Juliane Medler, Johannes Nelke, Daniela Weisenberger, Tim Steinfatt, Moritz Rothaug, Susanne Berr, Thomas Hünig, Andreas Beilhack, Harald Wajant

**Affiliations:** 1 Division of Molecular Internal Medicine, Department of Internal Medicine II, University Hospital Würzburg, Auvera Haus Grombühlstraße 12, 97080 Würzburg, Germany; 2 Department of Internal Medicine II, University Hospital of Würzburg, Zinklesweg 10, 97078 Würzburg, Germany; 3 Institute of Virology and Immunobiology, University of Würzburg, Versbacher Str. 7, 97078 Würzburg, Germany

## Abstract

Antibodies specific for TNFRSF receptors that bind soluble ligands without getting properly activated generally act as strong agonists upon FcγR binding. Systematic analyses revealed that the FcγR dependency of such antibodies to act as potent agonists is largely independent from isotype, FcγR type, and of the epitope recognized. This suggests that the sole cellular attachment, achieved by Fc domain-FcγR interaction, dominantly determines the agonistic activity of antibodies recognizing TNFRSF receptors poorly responsive to soluble ligands. In accordance with this hypothesis, we demonstrated that antibody fusion proteins harboring domains allowing FcγR-independent cell surface anchoring also act as strong agonist provided they have access to their target. This finding defines a general possibility to generate anti-TNFRSF receptor antibodies with FcγR-independent agonism. Moreover, anti-TNFRSF receptor antibody fusion proteins with an anchoring domain promise superior applicability to conventional systemically active agonists when an anchoring target with localized disease associated expression can be addressed.

## Introduction

Receptors of the tumor necrosis factor (TNF) receptor superfamily (TNFRSF) are naturally activated by ligands of the TNF superfamily (TNFSF)^1,2^. TNFSF ligands, with exception of LTα, are type II transmembrane proteins and are characterized by a C-terminal TNF homology domain (THD), which promotes assembly of homotrimeric molecules and binding to TNFRSF receptors^1,2^. Soluble TNFSF ligands occur naturally due to proteolytic processing in the stalk region separating the THD from the transmembrane domain but can also be generated by recombinant DNA technology^1,3^. Intriguingly, TNFRSF receptors differ in their reaction to binding of soluble ligand trimers. While signaling of one group of TNFRSF receptors is efficiently stimulated by binding of soluble ligand molecules, the receptors of a second group interact with soluble ligand trimers but are not or only poorly activated in this way^4^. Single TNFRSF receptor molecules bind to the grooves formed between the three protomers of a TNFSF ligand trimer, which results in the formation of symmetric complexes of one ligand trimer and three receptor molecules^4^. There is steadily growing evidence that a single TNFSF ligand trimer-TNFRSF receptor_3_ complex alone is insufficient to unleash the full signaling capacity of TNFRSF receptors. Instead, it appears that at least some TNFRSF receptor signaling pathways, e.g., the classical NFκB pathway and extrinsic apoptosis induction, require the secondary interaction of two or more trimeric TNFRSF receptor complexes^4^. In situations where membrane-bound TNFSF ligand trimers interact with equally membrane-restricted receptor molecules, the high local concentration of the molecules in the cell-to-cell contact zone and their reduced mobility are in any case sufficient to promote secondary clustering and full signaling. In the case of trimeric receptor complexes formed in response to soluble ligand binding, however, signaling-promoting secondary interaction may largely depend on a receptor type-specific intrinsic oligomerization capacity. Indeed, ligand-independent low affinity auto-aggregation has been demonstrated for several members of the TNFRSF^5^. Moreover, the poor response of TNFRSF receptors to soluble ligand trimers can be overcome by oligomerization (e.g., via antibody cross-linking or genetic fusion with oligomerization domains) or cell surface anchoring of the soluble ligand molecules^3,4^.

Therapeutic strategies aiming on the activation of TNFRSF receptors are difficult to realize. Recombinant soluble TNFSF ligands are often no option due to their poor receptor-stimulating activity and even when highly active soluble ligand variants are available their pharmacokinetics and production are typically challenging. Agonistic antibodies are therefore the means of choice when therapeutic TNFRSF receptor activation is envisaged. Preclinical and clinical studies in recent years with CD40-, CD95-, Fn14-, and TRAILR2/DR5-specific antibodies suggest that binding to FcγRs is a crucial step to unleash strong agonistic activity^6–15^. The resulting FcγR activation, however, can counteract or even negate the anticipated effect of the anti-TNFRSF receptor antibody, e.g., destruction of cells targeted with the aim to trigger immune stimulatory TNFRSF receptors.

Here, we show that antibodies targeting TNFRSF receptor types, which are not or only partially activated by soluble ligand molecules, possess regularly a strong FcγR-binding restricted agonistic activity, irrespective of their affinity and isotype and the epitope recognized. We further demonstrate that fusion proteins of such antibodies with an anchoring domain designed to bind to cells in an antigen- and FcγR-independent fashion are also highly agonistic provided they have access to their anchoring target. This finding allows the straightforward development of anti-TNFRSF receptor antibody fusion proteins with FcγR-independent agonistic activity. Moreover, it promises reduction of systemic side effects in cases where an anchoring target with localized disease associated expression can be addressed.

## Results

### Activation of a subgroup of TNFRSF receptors by IgG antibodies is strongly dependent on FcγR-binding

**Fig. 1:**
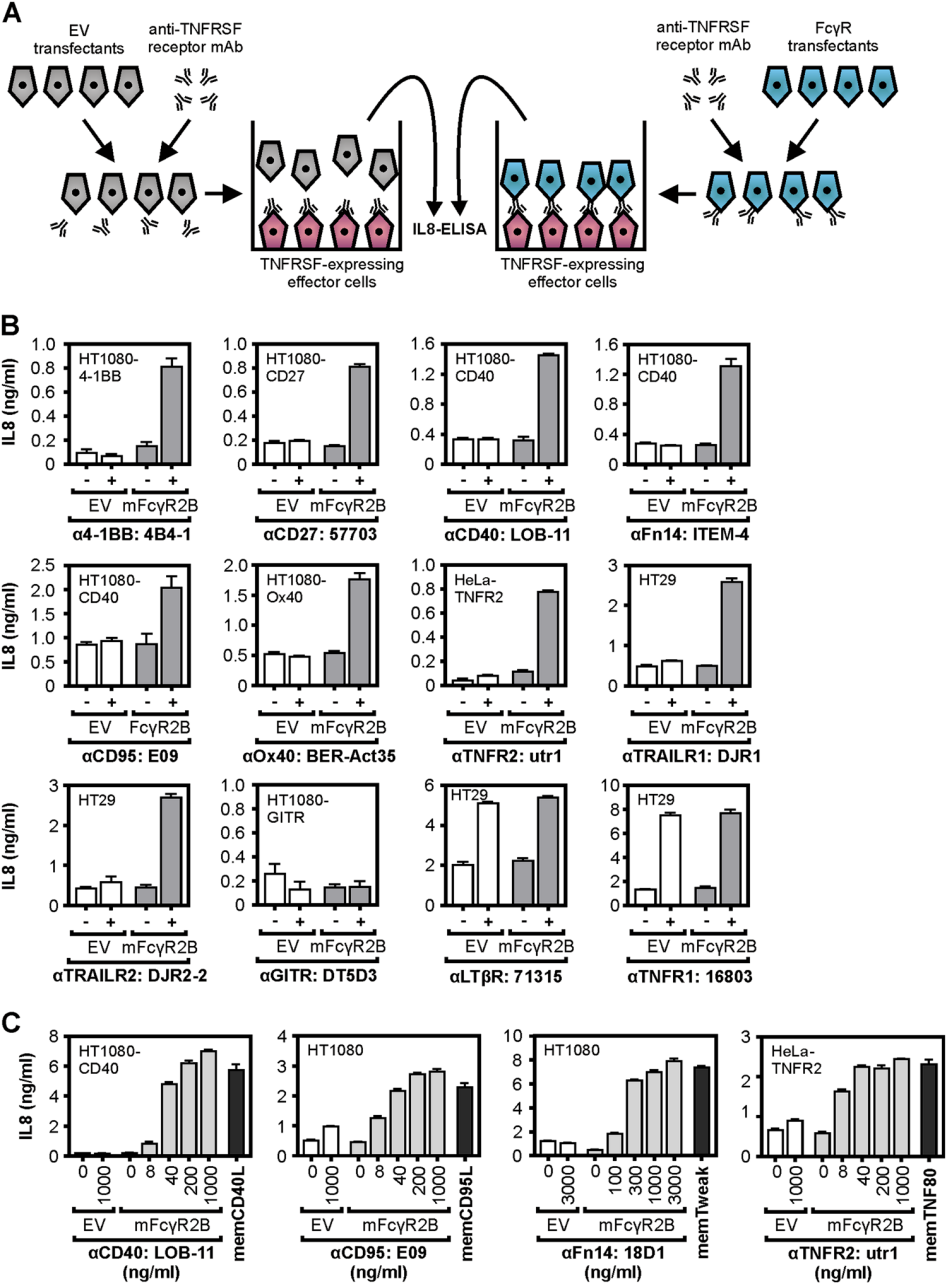
Murine FcγR2B enhances agonism of anti-TNFRSF receptor mIgG1 antibodies. **a** Scheme of coculture assay for the evaluation of FcγR-dependent agonism of TNFRSF receptor-specific antibodies. **b** The indicated TNFRSF receptor-responsible target cells were seeded in 96-well tissue culture plates. The following day, cells were challenged in triplicates with 300 ng/ml of the indicated anti-TNFRSF receptor antibodies and HEK293 cells transfected with empty vector (EV) or a murine FcγR2B-encoding expression plasmid. In case of the 4-1BB specific antibody 4B4-1 3000 ng/ml were used for stimulation. In contrast to the various TNFRSF receptor sensor cells, HEK293 cells have no or only low endogenous expression of the TNFRSF receptors studied and produce only limited amounts of IL8. After overnight incubation, cell supernatants were analyzed for IL8 production as readout of TNFRSF receptor activation. Death receptors were analyzed in the presence of the pan-caspase inhibitor ZVAD (20 µm) to prevent the disturbing effect of apoptosis induction. **c** TNFRSF receptor-responsible target cells were seeded and next day the cells were challenged in triplicates with increasing concentrations of the indicated anti-TNFRSF receptor antibodies and HEK293 cells transfected with empty vector (EV) or a murine FcγR2B-encoding expression plasmid. For comparison, the TNFRSF receptor-responsible target cells were cocultered with HEK293 cells transiently transfected with expression plasmids encoding the corresponding full-length ligand molecule. After overnight incubation IL8 in the supernatant were determined

Initially, we assessed the relevance of FcγR-binding of TNFRSF receptor-specific IgG antibodies for the agonism of these molecules in a broad and comprehensive manner. For this purpose, we analyzed at first a panel of ~30 murine antibodies recognizing more than 10 members of the TNFRSF in coculture assays of TNFRSF receptor-responsive target cells and empty vector (EV) or murine FcγR2B transfected HEK293 cells for their capacity to elicit TNFRSF receptor activation (Fig. 1a, b; Supplementary data Fig. S1). The antibodies were chosen based on their simple availability from the reagent stock of our research group and were originally acquired for quite different applications reaching from receptor blocking over receptor stimulation to FACS and ELISA applications (Supplementary data table S1). Since we were interested in the potential agonistic activity of the antibodies, we excluded, of course, antibodies recognizing the intracellular part of TNFRSF receptors or antibodies, which do not work in ELISA or FACS applications and which are therefore obviously not able to recognize the native receptor molecules. With exception of the decoy TNFRSF receptors, all receptors of the TNFRSF, including the death receptors (especially if apoptosis is blocked), activate the classical NFκB pathway. It is well established that transcription of the IL8/CXCL8 gene is induced via the classical NFκB pathway^16^. We therefore used measuring of IL8 induction as a convenient and sensitive method to detect TNFRSF receptor activation. All antibodies recognizing LTβR or TNFR1 induced strong IL8 production and showed thus significant agonistic activity. The observed agonistic activity was not or only modestly enhanced in the presence of murine FcγR2B-expressing cells (Fig. 1a, b; Supplementary data Fig. S1). With exception of one antibody against GITR, all other antibodies investigated, recognizing CD27, OX40, 4-1BB, TNFR2, CD40, CD95, TRAILR1, TRAILR2, or Fn14, displayed no or only modest agonistic activity but readily converted to potent receptor agonists in the presence of murine FcγR2B-expressing cells (Fig. 1a, b, Supplementary data Fig. S1). Thus, it appears that the type of TNFRSF receptor is the major factor, which determines whether agonistic antibody activity requires FcγR-binding or not. Interestingly, all TNFRSF receptors that required murine FcγR2B-binding of bivalent antibodies to become efficiently activated belonged to the subgroup of the TNFRSF which poorly respond to soluble ligand trimers (Table 1). Where investigated the maximal TNFRSF receptor-mediated IL8 response induced by FcγR-bound antibodies were in the range of those induced by transfectants expressing the corresponding membrane-bound TNFSF ligand (Fig. 1c).

**Table 1:** Activation of TNFRSF receptors by soluble TNFSF ligands and TNFRSF-specific antibodies

Receptor Type	Cellular response	Soluble ligand: EC50_w/o crosslinking_/ EC50_with crosslinking_	Antibody: EC50_w/o crosslinking_/ EC50_with crosslinking_
4-1BB	IL8	»100^28^	>10
CD27	IL8	»100^28^	>100
CD40	IL8	5–25^28^	>100
CD95	Cell death	100–»1000^37,38^	>100
Fn14	IL8	>100^32,37,39^	»100
GITR	IL8	~5^28^	Not detectable
LTßR	IL8	1^34^	1
Ox40	IL8	~100^29^	>100
TNFR1	Cell death	1^37^	1
TNFR2	Proliferation	Differ in max. response^37^	>100
TRAILR1	Cell death	70–»1000^40,41^	>100
TRAILR2	Cell death	70–»1000^40,41^	>100

### Agonistic activity of FcγR-bound anti-TNFRSF receptor antibodies is largely independent of isotype, FcγR type, and of the epitope recognized

**Fig. 2:**
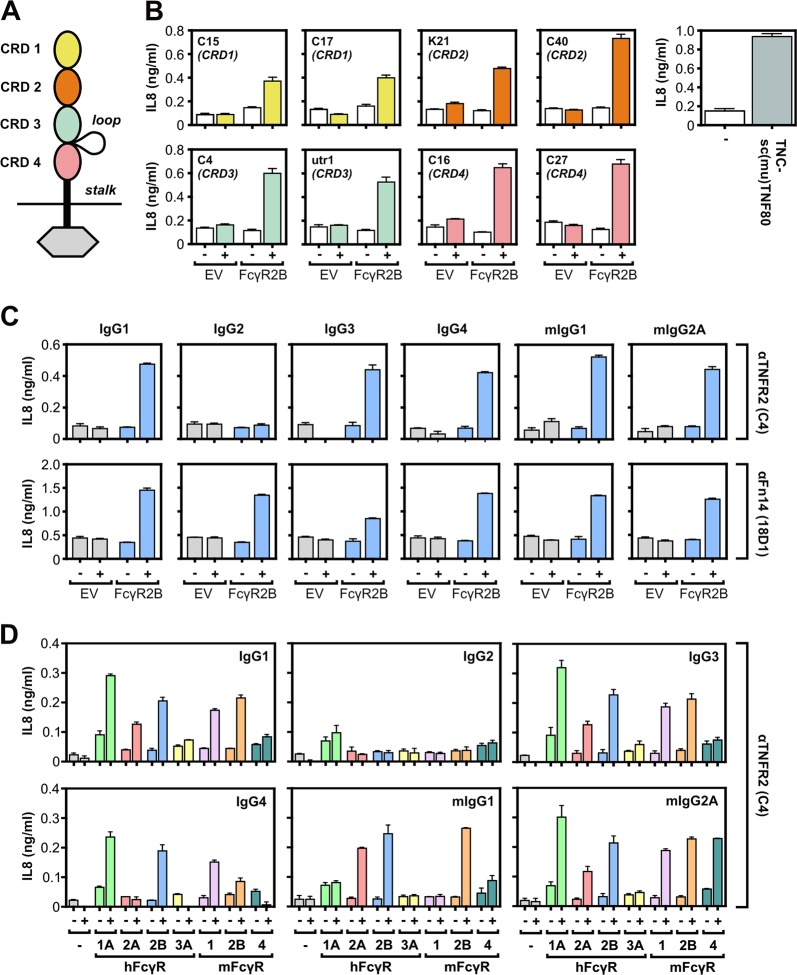
Isotype, FcγR type, and the recognized epitope are of secondary relevance for the FcγR-dependent agonism of anti-TNFRSF receptor antibodies. **a** Domain architecture of TNFR2. **b** HeLa-TNFR2 cells were incubated in triplicates with anti-TNFR2 antibodies recognizing the indicated domain of TNFR2 along with HEK293 cells transfected with empty vector (EV) or a FcγR2B-encoding expression plasmid. One day later, cell supernatants were analyzed for IL8 production. Stimulation with a saturating concentration (200 ng/ml) of the highly potent TNFR2 agonist TNC-scTNF80 served as a positive control. **c** TNFR2-responsive HeLa-TNFR2 cells and Fn14-responsive WiDr cells were challenged in triplicates with the indicated anti-TNFRSF receptor antibodies and empty vector (EV) or FcγR2B transfected HEK293 cells which have no (TNFR2) or only low (Fn14) endogenous expression of the TNFRSF receptors studied and which only produce limited amounts of IL8. Next day, cell supernatants were analyzed for IL8 production as readout of TNFRSF receptor activation. **d** HeLa-TNFR2 cells were treated overnight in triplicates with HEK293 cells transfected with empty vector or expression plasmids encoding the indicated FcγR types and 1 µg/ml of IgG1, IgG2, IgG3, IgG4, mIgG1, and mIgG2A variants of the anti-TNFR2 antibody C4. Finally, IL8 production was quantified by ELISA

To evaluate next the relevance of the epitope recognized for the FcγR dependency of the agonistic activity of anti-TNFRSF receptor antibodies, we investigated a group of human TNFR2-specific murine IgG1 antibodies. First, we identified antibodies that recognize the cysteine-rich domain 1 (CRD1), CRD2, CRD3, or CDR4 of TNFR2 by screening the interaction of a panel of GpL fusion proteins of soluble TNFR2 deletion mutants with the commercially available anti-TNFR2 utr-1 and a panel of in house produced anti-TNFR2 antibodies (Fig. 2a, Supplementary data Fig. S2). Eight antibodies, two for each of the four different CRDs of TNFR2, were then analyzed with respect to their TNFR2-stimulating activity in coculture assays of TNFR2-responsive HeLa-TNFR2 cells and HEK293 cells transiently transfected with EV or an expression plasmid encoding FcγR2B. All TNFR2-specific antibodies analyzed elicited at best low agonistic activity in the presence of EV transfected HEK293 cells but readily stimulated TNFR2 signaling in co-cultures with FcγR2B-expressing cells (Fig. 2b). This suggests that the epitope recognized by an anti-TNFRSF receptor IgG antibody is of only secondary relevance for its agonistic activity. To find out whether the isotype is of relevance for FcγR-dependent agonism of anti-TNFRSF receptor IgG antibodies, we generated and analyzed mIgG1, mIgG2A, IgG1, IgG2, IgG3, and IgG4 variants of the TNFR2-specific antibody C4 (this study) and of the Fn14-specific antibody 18D1^14^. All antibody variants alone turned out to be inactive or only poorly active (Fig. 2c). In the presence of the inhibitory FcγR FcγR2B, however, all antibody variants, with exception of the IgG2 variants, elicited strong TNFR2 and Fn14 signaling (Fig. 2c). With the IgG2 variant of 18D1, but not with those of C4, there was significantly enhanced agonistic activity in the presence of FcγR2B-expressing cells (Fig. 2c). This correlates to the fact that IgG2 has only low affinity for FcγRs^17^. A similar systematic investigation with a comprehensive panel of murine and human FcγRs and the various anti-TNFR2 antibody C4 isoforms revealed C4 isoform-specific enhancement patterns of TNFR2 agonism for the various FcγR types that correspond to their known antibody binding preferences (Fig. 2d). Thus, the isotype of an IgG antibody seems to be only of importance for the agonistic activity of anti-TNFRSF receptor antibodies as long it determines sole FcγR binding. To control that endogenous FcγR expression is sufficient to uncover strong agonism of anti-TNFRSF receptor IgGs, we performed coculture assays with TNFRSF receptor-responsive cells and THP-1 cells having endogenous FcγR expression (Supplementary data Fig. S3A,B). We investigated the Fn14-specific antibody Fn14 studied above and obtained similar results as before with the FcγR HEK293 transfectants (Supplementary data Fig. S3C).

**Fig. 3:**
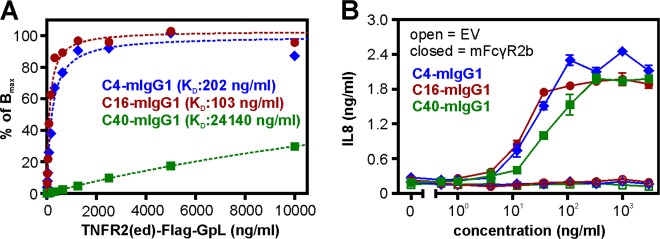
Antigen affinity and FcγR-dependent anti-TNFRSF receptor antibody agonism. **a** The affinities of the indicated TNFR2-specific mIgG1 antibodies were evaluated by binding studies with TNFR2-Fc-GpL and plastic immobilized antibodies. **b** Co-cultures of HeLa-TNFR2 cells and murine CD32B-expressing HEK293 cells were challenged overnight with the indicated concentrations of the TNFR2-specific mIgG1 antibodies. Finally, IL8 production was quantified by an ELISA

To judge the relevance of antigen affinity for FcγR-dependent agonism of anti-TNFRSF receptor antibodies, we performed comparative studies with three anti-TNFR2 antibodies whose affinity cover a range of two orders of magnitude (Fig. 3a). Despite the considerable differences in affinity all three antibodies stimulated TNFR2 signaling with a closely related dose response relation in the presence of murine FcγR2B-expressing cells (Fig. 3b) indicating that the FcγR-binding effect dominates over antigen affinity in the context of FcγR-dependent agonism of TNFR2-specific antibodies.

### The FcγR dependency of anti-TNFRSF receptor IgG agonism can be overcome by genetic fusion with heterologous cell surface anchoring domains

So far, our results consistently show that the sole anchoring to cell-expressed FcγRs is sufficient to confer strong agonistic activity to otherwise poorly active anti-TNFRSF receptor antibodies. Other factors that typically affect the outcome of antibody-antigen interaction, such as affinity, epitope specificity, isotype, and FcγR type, are of minor relevance. It is thus tempting to speculate that cell surface attachment per se is the decisive responsible factor conferring agonistic activity. To further test this hypothesis, we generated anti-TNFRSF receptor antibody fusion proteins which can interact with a defined cell surface-expressed anchoring target independent from FcγRs and the antigen recognized by the variable antibody domains.

**Fig. 4:**
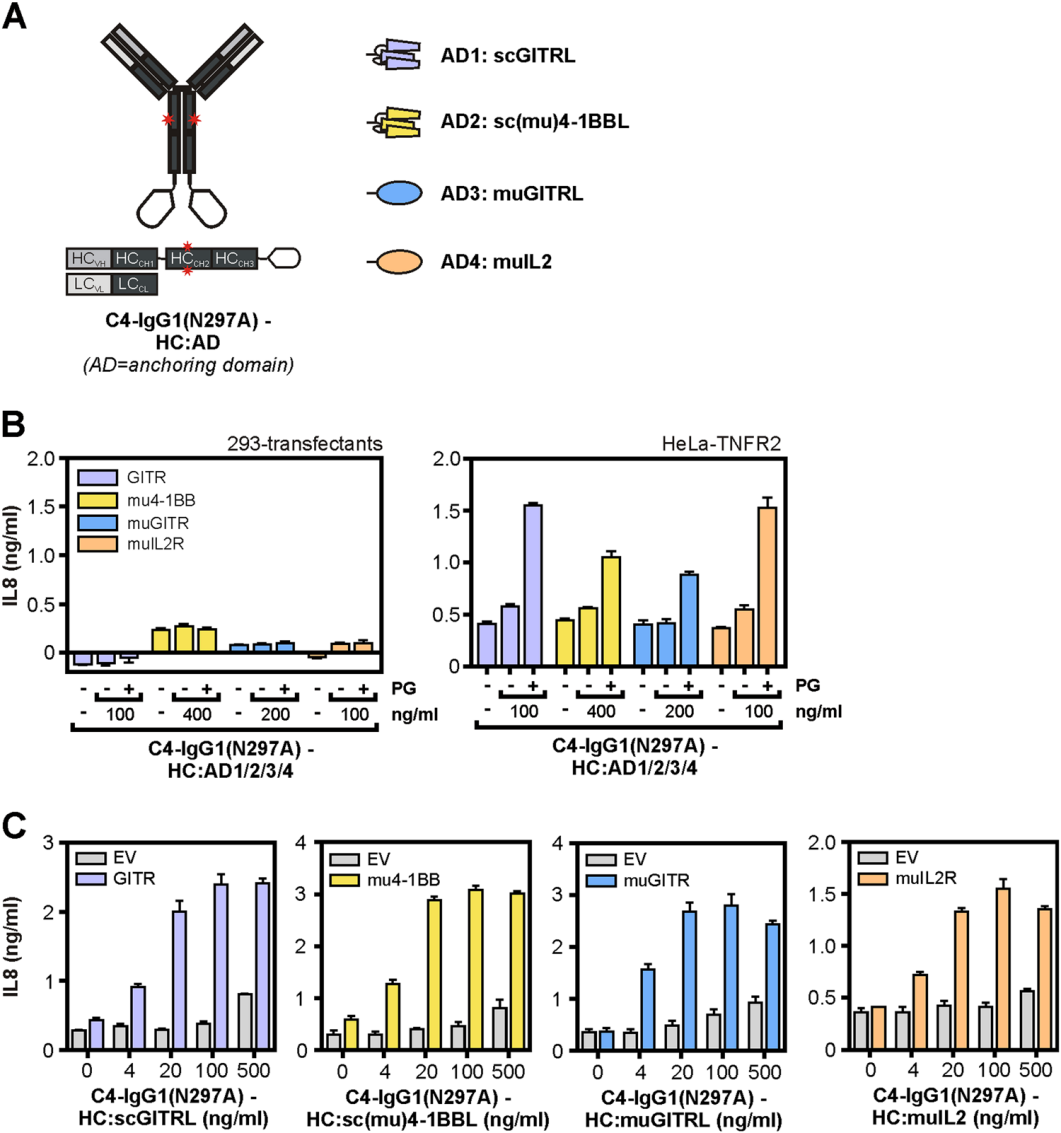
C4-IgG1(N297A) cytokine fusion proteins acquire high agonistic potential after cytokine receptor binding. **a** Domain architecture and structural organization of C4-IgG1(N297A)-HC:scGITRL, C4-IgG1(N297A)-HC:sc(mu)4-1BBL, C4-IgG1(N297A)-HC:(mu)GITRL, and C4-IgG1(N297A)-HC:muIL2. **b** HeLa-TNFR2 cells and HEK293 transfectants expressing the indicated cytokine receptors were stimulated with 200 ng/ml of the various C4-IgG1(N297A) cytokine fusion proteins in the presence and absence of 1 µg/ml protein G. Next day, IL8 content of supernatants were determined by ELISA. **c** Co-cultures of HeLa-TNFR2 cells and the various HEK293 transfectants were stimulated as indicated with the C4-IgG1(N297A) cytokine fusion proteins and IL8 production was again determined the following day by ELISA

In a first series of antibody fusion proteins, we genetically fused the C-terminus of the heavy chain of the TNFR2-specific IgG1 antibody C4-IgG1(N297A) with cytokines as anchoring domains allowing binding to corresponding cytokine receptor expressing cells. We used here a point mutant of IgG1 (N297A) that strongly interferes with binding to FcγR2A, FcγR2B, and FcγR3A^18^ (Supplementary data Fig. S4). To avoid/reduce self-aggregation of the antibody fusion proteins, we chose monomeric or dimeric cytokine variants. The following cytokine variants were used as anchoring domains: murine IL-2, murine GITRL, which form dimers, and single-chain encoded variants of the trimer-forming TNFSF ligands human GITRL and murine 4-1BBL, which act as monomeric binding domains (Fig. 4a). We performed coculture experiments with TNFR2-expressing HeLa cells and HEK293 cells transiently transfected with EV or expression plasmids encoding the subunits of the murine IL-2R, murine GITR, murine 4-1BB, and human GITR. Treatment of the cytokine receptor transfected HEK293 cells or the HeLa-TNFR2 alone with the four C4-IgG1(N297A) fusion proteins revealed no or only very moderate IL8 production (Fig. 4b). As expected after oligomerization with protein G all fusion proteins stimulated IL8 production by HeLa-TNFR2 cells (Fig. 4b, right panel). The various transfected HEK293 cells, however, produced minute amounts of this cytokine even in the presence of the protein G oligomerized antibody fusion proteins (Fig. 4b, left panel). Thus, in the described experimental setting IL8 production can be used again as a readout for TNFR2 activation. Stimulation of co-cultures of HeLa-TNFR2 cells and the human GITR expressing HEK293 transfectants with C4-IgG1(N297A)-HC:scGITRL resulted in strong IL8 production while there was only a minor effect on IL8 production of co-cultures of HeLa-TNFR2 and HEK293-EV cells (Fig. 4c). Similarly, C4-IgG1(N297A)-HC:sc(mu)4-1BBL, C4-IgG1(N297A)-HC:(mu)GITRL, and C4-IgG1(N297A)-muIL2 induced strong IL8 production in co-cultures of HeLa-TNFR2 cells with HEK293 cells transfected with mu4-1BB, muGITR, and muIL-2R complex encoding expression plasmids, respectively. Again these constructs showed no major effect on IL8 production of negative control HeLa-TNFR2/HEK293-EV co-cultures (Fig. 4c). This suggest that all four C4-IgG1(N297A) cytokine fusion proteins investigated have the ability to activate TNFR2 in an FcγR-independent manner upon anchoring to their corresponding cell surface exposed cytokine receptor. In accordance with this conclusion, the C4-IgG1(N297A) fusion proteins failed to trigger IL8 production in co-cultures where HeLa-TNFR2 cells have been replaced by conventional HeLa cells lacking TNFR2 expression (Supplementary data Fig. S5A). Similarly, preincubation of the various C4-IgG1(N297A) cytokine fusion proteins with soluble TNFR2-Fc inhibited the ability of these fusion proteins to induce IL8 production in co-cultures of HeLa-TNFR2 cells and HEK293 cells expressing the corresponding cytokine receptors (Supplementary data Fig. S5B).

**Fig. 5:**
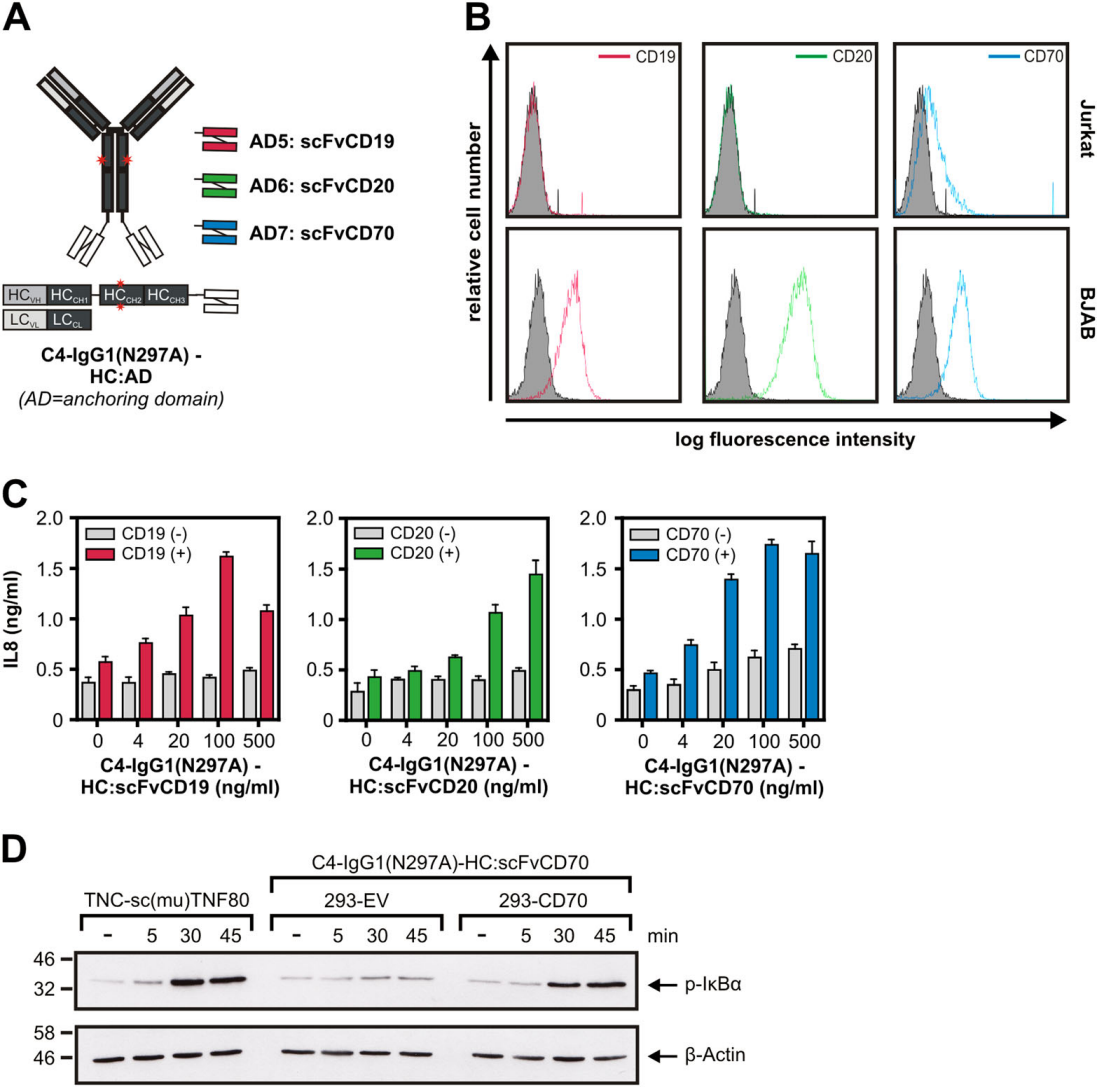
C4-IgG1(N297A) scFv fusion proteins acquire high agonistic potential after binding to the scFv-targeted cell surface antigen. **a** Domain architecture and structural organization of C4-IgG1(N297A)-HC:scFvCD19, C4-IgG1(N297A)-HC:scFvCD20, and C4-IgG1(N297A)- HC:scFvCD70. **b** Analysis of CD19, CD20, and CD70 expression of Jurkat and BJAB cells by FACS. **c** HeLa-TNFR2 cells and BJAB (CD19^+^ CD20^+^ CD70^+^) or Jurkat cells were cocultured and stimulated overnight with the indicated concentration of the various C4-IgG1(N297A) scFv fusion proteins. IL8 content of supernatants were determined by ELISA. **d** HeLa-TNFR2 cells alone or in combination with the indicated HEK293 transfectants were preincubated with 20 µM MLN4924 for 30 min and afterwards stimulated for the different times with C4-IgG1(N297A)-HC:scFvCD70 or TNC-sc(mu)TNF80, a potent TNFR2 agonist as a positive control. MLN4924 was used to prevent proteasomal IκBα degradation to rule out underestimation of IκBα phosphorylation. MLN4924 is an inhibitor which interferes with the activity of the E3 ligase complex responsible for K48 ubiquitination of IκBα

In a second series of experiments, we evaluated C4-IgG1(N297A) fusion proteins with cell surface antigen-specific scFvs as anchoring domain. Specifically, we genetically fused scFvs specific for the tumor-associated antigens CD19, CD20, and CD70 to the C-terminus of the C4-IgG1(N297A) heavy chain (Fig. 5a). There was no or only poor IL8 production by the three C4-IgG1(N297A)-HC:scFv fusion proteins in HeLa-TNFR2 cells cocultured with Jurkat cells having no or only very low expression of CD19, CD20, and CD70 (Fig. 5b). The presence of the scFv anchoring domain-specific targets expressing BJAB cells lowered the EC50 values of the C4-IgG1(N297A)-HC:scFv fusion proteins for IL8 induction >100 fold (Fig. 5c). To demonstrate stimulation of TNFR2-induced NFκB signaling by a cell surface target-anchored C4-IgG1(N297A)-HC:scFv fusion protein with a second independent method, we analyzed the ability of CD70-anchored C4-IgG1(N297A)-HC:scFvCD70 to trigger IκBα phosphorylation, a hallmark event of the classical NFκB pathway. As before, with cocultured CD70-negative cells there was no evidence for TNFR2 activation. In contrast, in the presence of CD70-expressing cells there was again strong TNFR2 signaling by C4-IgG1(N297A)-HC:scFvCD70 (Fig. 5d). Thus, similar to the C4-IgG1(N297A) cytokine fusion proteins, the C4-IgG1(N297A)-HC:scFv fusion proteins showed superior agonism upon cell anchoring.

**Fig. 6:**
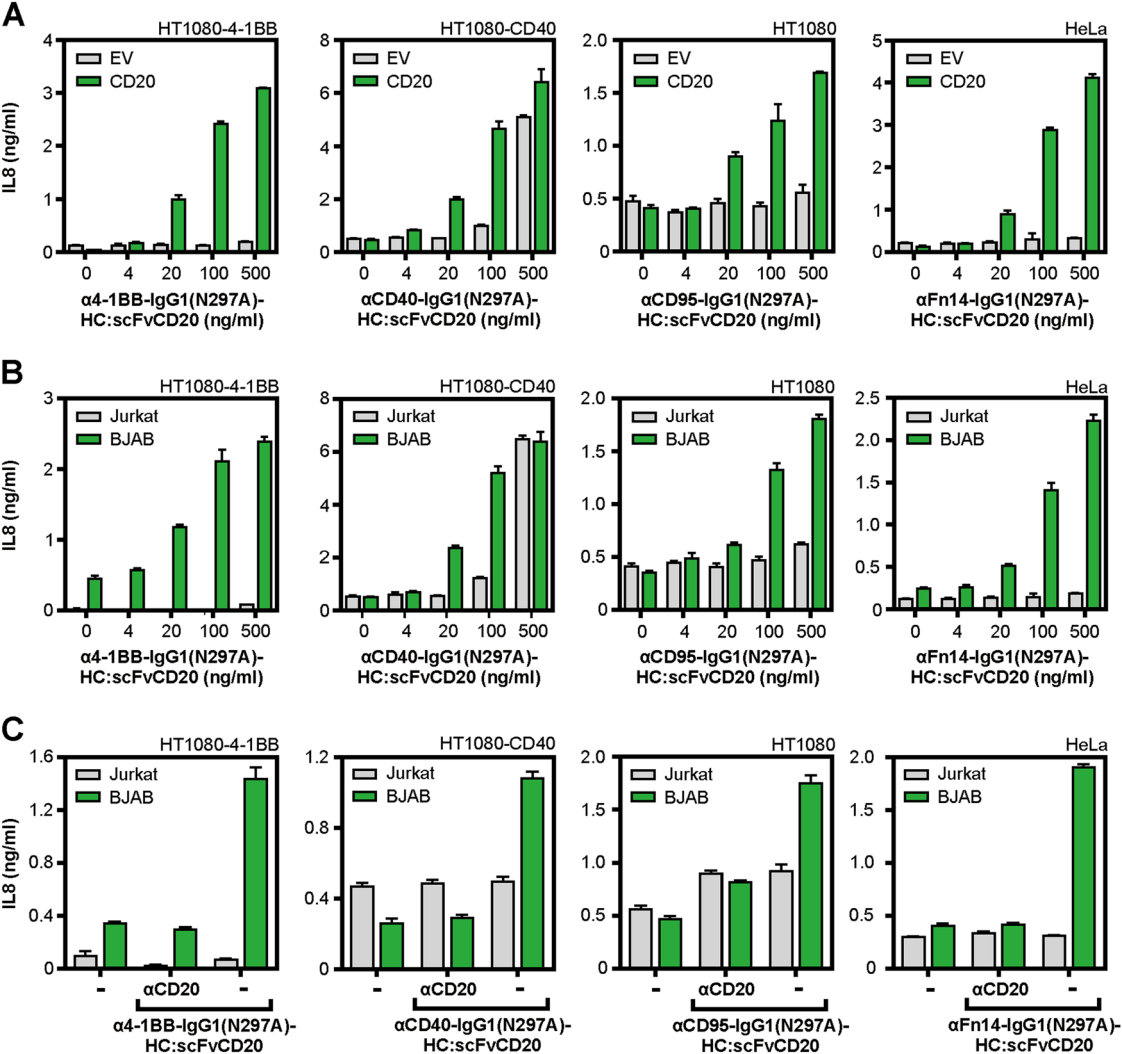
4-1BB-, CD40-, CD95-, and Fn14-specific IgG1(N297A)-HC:scFvCD20 fusion proteins display CD20-restricted agonism. **a** CD40- (HT1080-CD40), 4-1BB- (HT1080-4-1BB), CD95- (HT1080), and Fn14-responsible cells (HeLa) were cocultured with HEK293-EV and HEK293-CD20 cells. Co-cultures were stimulated with the indicated concentrations of anti-4-1BB-IgG1(N297A)-HC:scFvCD20, anti-CD95-IgG1(N297A)-HC:scFvCD20, anti-Fn14-IgG1(N297A)-HC:scFvCD20, and anti-CD40-IgG1(N297A)-HC:scFvCD20. Next day, TNFRSF receptor activation was evaluated by measuring IL8 production. The CD95-specific antibody was applied in the presence of 20 µM ZVAD to prevent apoptosis. **b** Co-cultures were performed as described in “A” with the difference that HEK293-EV cells have been replaced by Jurkat cells and HEK293-CD20 cells by BJAB cells. **c** Co-cultures of Jurkat or BJAB cells with the indicated TNFRSF receptor-responsive cell lines were stimulated overnight in the presence and absence of 50 µg/ml anti-CD20-IgG1 with 100 ng/ml of the various IgG1(N297A)-HC:scFvCD20 fusion proteins and IL8 production was finally quantified by ELISA. In case of anti-CD40-IgG1(N297A)-HC:scFvCD20 cells were treated with only 20 ng/ml

To find out whether cell surface binding of antibodies with the help of a genetically fused anchoring domain represents a broadly applicable strategy to yield highly agonistic anti-TNFRSF receptor antibody fusion proteins with FcγR-independent activity, we constructed and evaluated a panel of CD20-anchoring IgG1-scFv fusion proteins using the 4-1BB-specific antibody HBBK4 (WO 2006/126835 A1), the CD40-specific antibody G28.5^19^, the CD95-specific antibody E09^20^, and the Fn14-specific antibody 18D1^14^. In coculture experiments with HEK293-EV cells and 4-1BB, CD40, CD95, and Fn14 responsible cells, there was no evidence for agonistic activity of anti-4-1BB-IgG1(N297A)-HC:scFvCD20, anti-CD95-IgG1(N297A)-HC:scFvCD20, and anti-Fn14-IgG1(N297A)-HC:scFvCD20 up to 500 ng/ml (Fig. 6a). In the case of anti-CD40-IgG1(N297A)-HC:scFvCD20 there was significant CD40-mediated IL8 production but only at the highest concentration of 500 ng/ml (Fig. 6a). In co-cultures with CD20 transfected HEK293 cells, however, all IgG1-scFv fusion proteins triggered IL8 production, thus TNFRSF receptor activation, starting at concentrations around 20 ng/ml (Fig. 6a). Similar results were obtained in co-cultures where HEK293-EV and HEK293-CD20 transfectants have been replaced by Jurkat cells which do not express CD20 and BJAB cells which endogenously express CD20 (Fig. 6b). Blockade of CD20 using high concentrations of the anti-CD20 antibody Rituximab abrogated the agonistic activity of the IgG1-scFv fusion proteins emphasizing the dependency of agonistic activity from cell surface anchoring (Fig. 6c). We furthermore investigated the ability of a cell surface target-anchored anti-CD95 fusion protein to trigger apoptosis. For this, we used HeLa cells which have no endogenous TNFR2 expression and HeLa-TNFR2 transfectants to exploit TNFR2 as an anchoring target. The anchoring domain of the TNFR2-targeted anti-CD95 fusion protein consisted of three peptide linked copies of a TNFR2-specific TNF mutant (Supplementary data Fig. S6A^21^). TNC-sc(mu)TNF80, a potent murine TNF-based agonist for human and murine TNFR2^22^, and the parental anti-CD95-IgG1(N297A) antibody showed no or only very moderate cytotoxic activity on both HeLa and HeLa-TNFR2 cells (Supplementary data Fig. S6B,C). anti-CD95-IgG1(N297A)-HC:sc(mu)TNF80, however, elicited strong activation of caspases and induced cell death at very low concentrations on HeLa-TNFR2 cells but was poorly cytotoxic on HeLa cells. This again demonstrated that cell surface anchoring of anti-CD95-IgG1(N297A) is sufficient to confer potent CD95 agonism. Similar results were obtained with anti-CD95-IgG1(N297A)-HC:scFvCD20 and coculture assays of HeLa cells with CD20-negative and CD20-expressing cells (Supplementary data Fig. S6D-F).

Using the Fn14-specific antibody 18D1 and the scFvCD20 anchoring domain as an example, we finally evaluated whether the isotype of the antibody part of an antibody fusion protein is of relevance for the anchoring domain-dependent agonism of the latter. Besides anti-Fn14-IgG1(N297A)-HC:scFvCD20, anti-Fn14-IgG2-HC:scFvCD20, anti-Fn14-IgG3-HC:scFvCD20, and anti-Fn14-IgG4-HC:scFvCD20, we also investigated a Fab_2_ variant of 18D1 with the scFvCD20 domain on the C-terminus of the truncated heavy chain (Supplementary data Fig. S7A). In coculture experiments of Fn14-responsive WiDr cells with HEK293-EV and HEK-CD20 transfectants cells, all anti-Fn14-IgG-HC:scFvCD20 fusion proteins and anti-Fn14-Fab_2_-HC:scFvCD20 revealed strong agonism in the HEK293-CD20 co-cultures but remained practically inactive in the HEK293-EV co-cultures (Supplementary data Fig. S7B). Similar results were obtained with WiDr co-cultures with CD20-positive BJAB cells and CD20-negative Jurkat cells (Supplementary data Fig. S7C). These data emphasize again that the sheer cell surface anchoring of anti-TNFRSF receptor fusion proteins is sufficient to overcome the FcγR dependency of the “parental” antibody. Worth mentioning, the genetic fusion of an anchoring domain to IgG1 or the IgG1(N297A) variant showed no significant effect on the affinity for the neonatal Fc receptor FcRn (Supplementary data Fig. S8). Thus, one could expect that anti-TNFRSF receptor fusion proteins have similar good pharmacokinetics as their conventional counterparts.

## Discussion

Due to the central role of TNFSF ligands and TNFRSF receptors in immune regulation, these molecules have attracted overwhelming interest as targets in the therapy of immune diseases and cancer^23^. Inhibition of TNFRSF receptors but also activation of TNFRSF receptors represent promising treatment options^23^. Inhibitory approaches can be straightforwardly realized by ligand neutralizing antibodies as it is illustrated by the clinical success of TNF- and RANKL-blocking antibodies^24–26^. TNFRSF receptor-stimulatory antibodies, however, have not entered yet clinical practice although a variety of supposed agonistic anti-TNFRSF receptor antibodies have been broadly evaluated in preclinical studies and clinical trials^23^. Notably, the huge majority of antibodies investigated targets CD40, 4-1BB, OX40, CD27, TNFR2, Fn14, CD95, and the TRAIL death receptors, which all belong to the subgroup of the TNFRSF which is not or only poorly activated by soluble ligand or antibody molecules. For antibodies targeting this type of TNFRSF receptors there are three major limitations: (i) lack of agonistic activity in vivo due to insufficient FcγR-binding, (ii) induction of therapy counteracting effects via the FcγRs required to unleash their full agonistic potential, and (iii) dose limiting side effects due to systemic activation of the targeted TNFRSF receptor type^9–12,15^.

We found a broadly and generally applicable method solving all three problems: genetic tagging of the anti-TNFRSF receptor antibody of interest with an anchoring domain enabling FcγR-independent binding to the cell surface. Cell surface targeting of such antibody fusion proteins via their anchoring domain uncovers an agonistic activity comparable to those reached upon FcγR binding. Thus, the sheer cell surface attachment of the anti-TNFRSF receptor antibody, irrespective of how this is reached, is obviously sufficient to unleash agonistic activity. Accordingly, affinity, isotype and the recognized epitope of an anti-TNFRSF receptor antibody had no major effect on FcγR-dependent agonism (Figs. 2, 3). Similarly, the specific features of the antibody part and the nature of the anchoring domain turned out to be largely irrelevant for the cell surface targeting-mediated agonism of anti-TNFRSF receptor antibody fusion proteins (Figs. 4–6, S5B). Thus, the decisive factor determining the agonistic potential of cell surface anchoring antibody fusion proteins is the anchoring domain and its target while there are no particular requirements on the antibody part. The choice of an appropriate useful anchoring domain is of course dependent on the intended therapeutic aim. For example, in immune oncological approaches the use of tumor antigen-specific scFvs as anchoring domains not only makes FcγR-binding superfluous but also promises to reduce systemic side effects. Since tumor-associated antigens can reach much higher expression levels as compared to FcγRs, it is tempting to speculate that cell surface-anchored anti-TNFRSF receptor antibody fusion proteins can even gain higher total activity than FcγR-bound conventional anti-TNFRSF receptor antibodies. The relative freedom in the choice of the anchoring domain offers furthermore the possibility to introduce a second effector function besides those of the antibody part. For example, the use of cytokines as anchoring domains links TNFRSF activation with cytokine receptor activation (Figs. 4, S5). Likewise, the use of scFvs that block tumor relevant receptors/ligands as anchoring domains may link TNFRSF activation with inhibition of protumoral activities.

It might be surprising that the agonistic activity of FcγR- and cell surface target-anchored antibodies and antibody fusion proteins is largely independent of the epitope recognized in a TNFRSF receptor. We suggest the following explanation: there is growing evidence reviewed in ref. ^4^ that (i) TNFRSF receptors interact in a 1:1 stoichiometry with adapter proteins (or protomers of trimeric adapter proteins) and that (ii) two TNFRSF receptor trimers (and thus six adapter proteins) are required to form intracellular signaling engaging adapter protein platforms. Thus, cross-liking by protein G or the enforced neighborhood of anchored antibodies in the cell-to-cell contact zone is necessary to bring the three or more (TNFRSF receptor)_2_-IgG1 complexes together which are required for the formation of the signaling competent hexameric/oligomeric TNFRSF receptor clusters. Since only the number of receptors/adapter molecules (≥6) and the proximity of the cytoplasmic domains is of relevance in this model, there is no special requirement for the epitope recognized by the antibody.

## Methods

### Cell lines and reagents

BJAB, HEK293, HeLa, HT1080, HT29, Jurkat, Raji THP-1, and WiDr cells (ATCC, Rockville, MD, USA) as well as the stable transfected HeLa-TNFR2 cells^27^ and the different HT1080-transfectants^28,29^ were cultivated in RPMI1640 medium (Sigma-Aldrich, Steinheim, Germany) supplemented with 10% fetal calf serum (GIBCO, EU Approved, South America). Human und murine FcγR encoding pCMV-SPORT6 expression plasmids were purchased from SourceBioscience. Expression plasmids for full-length, thus membrane-bound TNFSF ligands have been described elsewhere (CD95L^30^, TNFR2-specific TNF(143 N/145 R)^31^, TWEAK^32^) or was obtained by cloning of a corresponding PCR amplicon into pEYFP-C1 (Clontech Laboratories, Inc.).

For FACS analysis of CD19, CD20, and CD70 expression FITC-labeled antibodies from BD Bioscience, San Jose, CA, USA (anti-CD19 FITC-labeled mouse IgG1, #345776; anti-CD70 FITC-labeled mouse IgG3, #555834), Caprico Biotechnologies, Norcross, GA, USA (anti-CD20 FITC-labeled mouse IgG1, #103714), and Miltenyi Biotec GmbH, Bergisch Gladbach, Germany (anti-murin IgG1 FITC-labeled rat IgG1, #130-095-897) were used. Antibodies for evaluation of FcγR expression on THP-1 cells antibodies were from Santa Cruz Biotechnology, Santa Cruz, CA, USA (anti-CD16 PE-labeled mouse IgG1, #sc-19620; anti-CD64 PE-labeled mouse IgG1, #sc-1184; anti-CD32A/C mouse IgG1, #sc-373721; anti-CD32B mouse IgG1, #sc-365864) and Sigma, Saint Louis, MO, USA (anti-mouse IgG PE-labeled polyclonal goat, #P9670). Following primary antibodies have been used for western blotting: anti-caspase-8 (Enzo, 5F7), anti-caspase-9 (Cell Signaling, #9502), anti-CYLD (Cell Signaling, D1A10), and anti-phospho-IκBα (Ser32) (Cell Signaling, 14D4). HRP-labeled secondary antibodies were from Cell Signaling (anti-rabbbit (#7074)) or Dako (anti-mouse (#P0260). Detailed information about the antibodies used for TNFRSF receptor-activation studies are summarized in table S1. Humira^®^ (anti-TNF) Enbrel^®^ (TNFR2-Fc), and Rituximab^®^ (anti-CD20) were a kind gift of Prof. Dr. Hans-Peter Tony (Department of Internal Medicine II, University Hospital Würzburg).

### Generation of anti-TNFR2 monoclonal antibodies

TNFR2 knockout mice were immunized two times with a mixture of 50 µg TNFR2-Fc (Enbrel) and 50 µg muTNFR2(ed)-F-Fc i.p. (day 0 and 28). Three days after an additional i.v. injection of 10 µg per antigen on day 49 splenic cells were fused with X63-Ag8.653 cells using polyethylene glycol. The supernatants produced by the hybridoma cells were analyzed for binding to human and murine TNFR2 by flow cytometry and binding studies as described below. Positive clones for binding one or both of the antigens were subcloned by limiting dilution.

### Cloning and production of antibodies and antibody fusion proteins

The expression plasmids required to produce the various antibodies and antibody fusion proteins were cloned using standard techniques and appropriate synthetic DNA fragments or PCR amplicons and encoded for the protein sequences indicated in the Supplementary table S2. For production of the various antibodies and fusion proteins, HEK293 were transiently transfected with 1:1 mixtures of the corresponding light and heavy chain variant by using the transfection reagent PEI (polyethylenimine; Polyscience Inc., Warrington, USA) as described elsewhere^33^. The concentration of the produced fusion proteins was determined by SDS-PAGE and anti-Flag western blotting in comparison to a Flag-tagged standard protein^29^.

### FACS analysis

For analysis of cell surface expression of proteins, 5 × 10^5^ cells were washed twice with PBS and were subsequently incubated for 30 min at 4 °C with FITC- or PE-labeled antibodies of the specificity of interest. After removal of unbound antibodies by washing the cells three times with PBS, cells were finally analyzed using FACSCalibur (BD Biosciences, Heidelberg, Germany) following standard procedures.

### IL8-ELISA

For evaluation of the TNFRSF receptor-stimulatory potential of the various anti-TNFRSF receptor antibodies and antibody fusion proteins, cells (2 × 10^4^ cells/well; 96 tissue culture plates) strongly responsive to the TNFRSF receptor type of interest were seeded in triplicates in RPMI1640 medium supplemented with 10% FCS. Next day, medium was exchanged with fresh RPMI1640/FCS medium containing anchoring target positive or negative cells (2 × 10^4^/well), which do not or poorly respond to the TNFRSF receptor type investigated. Co-cultures were then challenged overnight with the antibodies and antibody fusion proteins. IL8 production, as an established and simple readout for activation of all TNFRSF receptors investigated in our study [e.g., refs. ^28,29,31,34,35^], was finally quantified by help of a ELISA kit according to the manufactures instruction (BD Biosciences, San Diego, USA). In the case of evaluation of apoptosis inducing TNFRSF receptors (CD95, TRAILR1, TRAILR2) cells were additionally treated with 20 µM ZVAD (Bachem, Heidelberg, Germany) to prevent cell death induction. In some experiments, anchoring target positive cells were omitted and replaced by co-treatment with 1 µg/ml protein G (Merck, Darmstadt, Germany).

### Viability assay

To evaluate effects on cellular viability, cells were seeded and stimulated as in the experiments for measuring IL8 induction. Where indicated, cells were pretreated with 2 µg/ml CHX. To normalize the viability of cells an additional group was stimulated with a cytotoxic mixture containing 0.02% sodium azide and cycloheximide (50 µg/ml) to define complete cell death. After overnight incubation the cell viability was determined by crystal violet staining.

### Binding studies

To identify the region of the extracellular domain of TNFR2 that is recognized by the various anti-TNFR2 antibodies, black high bond 96-well ELISA plates were coated with 1 µg/ml of anti-mouse IgG Fc (R&D Systems, Minneapolis, MN, USA). After overnight incubation at 4 °C the plates were washed three times with PBS and remaining free binding sites were blocked with 10% FCS in PBS (1 h, RT). TNFR2-specific antibodies (5 µg/ml) were then added for 2 h at RT and unbound molecules removed by three washes with PBS. The immobilized anti-TNFR2 antibodies were incubated (1 h, RT) with 2 µg/ml of a series of C-terminal deletion mutants of TNFR2 harboring a C-terminal *Gaussia princeps* luciferase (GpL) domain (TNFR2(ed)-GpL: TNFR2(aa:23-257 ac. no. NP_001057.1)-AGEF-Flag-(aa:63–230 ac. No. AEX33289.1); TNFR2(ed)-Δstalk-GpL: TNFR2(aa:23–201 ac. no. NP_001057.1)-Flag-Flag-(aa:63–230 ac. No. AEX33289.1); TNFR2(ed)-ΔCRD2–4-GpL: TNFR2(aa:23–76 ac. no. NP_001057.1)-Flag-(aa:63–230 ac. No. AEX33289.1); TNFR2(ed)-ΔCRD3-4-GpL: TNFR2(aa:23–119 ac. no. NP_001057.1)-Flag-Flag-(aa:63–230 ac. No. AEX33289.1); TNFR2(ed)-Δloop + CRD4-GpL:TNFR2(aa:23–144 ac. no. NP_001057.1)-Flag-Flag-(aa:63–230 ac. No. AEX33289.1); and TNFR2(ed)-ΔCRD4-GpL:TNFR2(aa:23–160 ac. no. NP_001057.1)-Flag-Flag-(aa:63–230 ac. No. AEX33289.1). Finally, plates were washed five times with PBS and the remaining anti-TNFR2 bound TNFR2 deletion mutant molecules were quantified by help of their GpL domain and the Gaussia luciferase Assay Kit (New England Biolabs GmbH, Frankfurt a. M., Germany) according to the recommendations of the supplier. The affinity of anti-TNFR2 mIgG1 antibodies to TNFR2 was determined in a related fashion using different concentrations of the GpL-tagged extracellular domain of TNFR2 and fitting of the obtained binding data to a one side specific binding plot using GraphPad Prism5. Non-specific binding values were obtained from anti-mouse IgG Fc coated wells incubated with mIgG1 of irrelevant specificity.

To determine the affinity of αFn14-IgG1-LC:GpL for the FcγRs expressed on THP-1 cells, equilibrium binding studies were performed. 5 × 10^5^ THP-1 cells were incubated with increasing concentrations of αFn14-IgG1-LC:GpL in the presence (unspecific binding) and absence (total binding) of an irrelevant IgG1 mixture. After incubation for 90 min at 37 °C, cells were washed three times with ice-cold PBS and then transferred to a black 96-well plate. GpL activity was again measured by use of the *Gaussia luciferase* Assay Kit and *K*_D_-values were calculated by fitting the binding data to a one side specific binding plot using GraphPad Prism5. To determine the affinity of various anti-CD95 fusion protein variants for FcRn equilibrium binding studies with HEK293 cells transfected with EV (unspecific binding) or a mixture of expression plasmids encoding FcRn and the subunit ß2-microglobulin (total binding) were performed. The assay procedure was the same as described above with exception that media and buffer with pH 5,5 have been used^36^.

### Western blot analysis

For western blot analysis 8 × 10^5^ HeLa or HeLa-TNFR2-cells were seeded in 6-well plates and stimulated on the next day as indicated. SDS-polyacrylamide gel electrophoresis and western blotting were performed as described previously^31^.

## Supplementary information

supplemental figures

supplemental tables
