# Supplementary material for: TNFRSF receptor-specific antibody fusion proteins with targeting controlled FcγR-independent agonistic activity
Source: Cell Death Dis. 2019 Mar 4;10(3):224. doi: 10.1038/s41419-019-1456-x (PMC6399339; doi:10.1038/s41419-019-1456-x)
Supplement: Supplementary file 1 — supplemental figures [file 41419_2019_1456_MOESM1_ESM.pdf]

Supplemental Figure 1

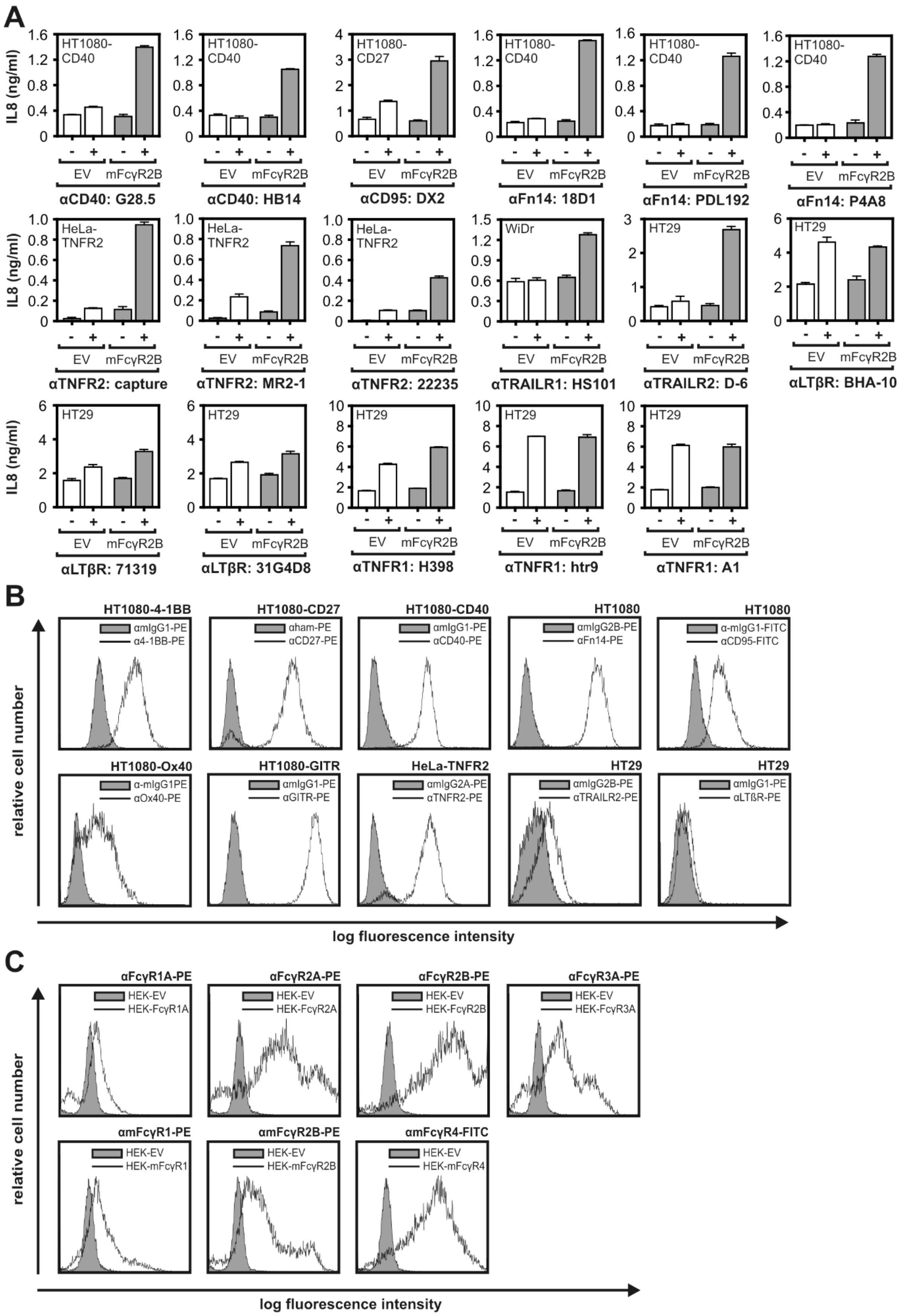

Figure legend, see next page.

Fig. S1. (A) The indicated TNFRSF receptor-responsible target cells were challenged in triplicates with 300 ng/ml of the indicated anti-TNFRSF receptor antibodies. HEK293 cells transfected with empty vector (EV) or a murine FcγR2B-encoding expression plasmid were added and the next day IL8 production was determined to evaluate TNFRSF receptor activation. Death receptor-specific antibodies were analyzed in the presence of ZVAD (20 µm) to prevent the disturbing effect of apoptosis induction. (B) The stable transfectants and cell lines were analyzed by flow cytometry for the expression of the indicated TNFRSF receptors. (C) Representative flow cytometry analysis of Hek293 cells transiently transfected with expression plasmids encoding the indicated Fc receptors.

Following antibodies were used for flow cytometry: anti-mFcγR1-PE labeled rat IgG2A (R&D Systems, Minneapolis, MN, USA; # FAB20741P), anti-mFcγR2B-PE labeled rat IgG2A (Milteny Biotec, Bergisch Gladbach, Germany; #130-092-572), anti-mFcγR4-FITC labeled rabbit IgG (Sino Biological, Wayne, PA, USA; #50036-R012-F), anti-4-1BB-PE labeled mouse IgG1 (BD Bioscience, San Jose, CA, USA; #555956), anti-CD27-PE labeled hamster IgG (eBioscience, San Diego, CA, USA; #12-0271-82), anti-CD40-PE labeled mouse IgG1 (Milteny Biotec, Bergisch Gladbach, Germany; #130-094-135), anti-Fn14-PE labeled mouse IgG2B (eBioscience, San Diego, CA, USA; #12-9018-82), anti-CD95-FITC labeled mouse IgG1 (R&D Systems, Minneapolis, MN, USA; # FAB142F), anti-OX40-PE labeled mouse IgG1 (BD Bioscience, San Jose, CA, USA; #558164), anti-GITR-PE labeled mouse IgG1 (Milteny Biotec, Bergisch Gladbach, Germany; # 130-092-895), anti-TNFR2-PE labeled mouse IgG2A (R&D Systems, Minneapolis, MN, USA; # FAB226P), anti-TRAILR2-PE labeled mouse IgGB (R&D Systems, Minneapolis, MN, USA; #FAB6311P), anti-LTßR-PE labeled mouse IgG1 (R&D Systems, Minneapolis, MN, USA; #FAB629P). Isotype controls were from R&D Systems, Minneapolis, MN, USA (anti mouse IgG1-FITC #555748; anti mouse IgG2A-PE #IC003P; anti mouse IgG2B-PE #IC0041P) and Santa Cruz, Dallas, TX, USA (anti hamster IgG-PE #sc2875).

Supplemental Figure 2

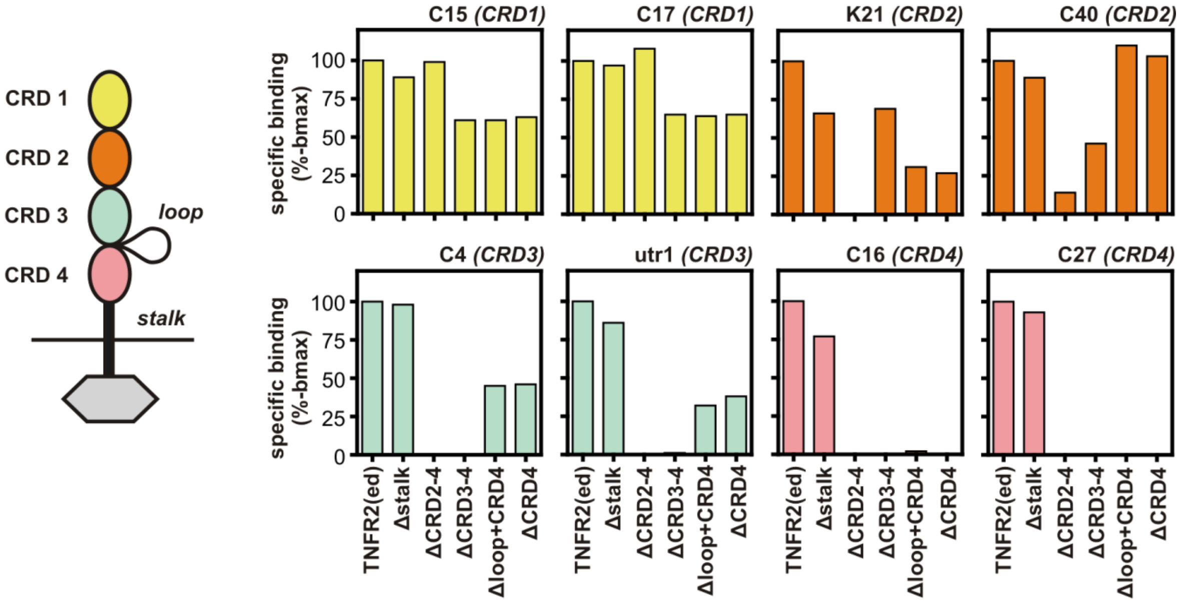

Fig. S2. Black high bond 96-well ELISA plates were coated with 1 µg/ml of anti-mouse IgG-Fc. After blocking the indicated TNFR2-specific antibodies (C15, C17 etc.) were added with 5 µg/ml for 2 h and unbound molecules were removed. The anti-Fc/anti-TNFR2 complexes were incubated (1 h, RT) with a series of C-terminal deletion mutants of TNFR2 (2 µg/ml) lacking the indicated parts and harboring a C-terminal *Gaussia princeps* luciferase (GpL) domain. Bound TNFR2 deletion mutant molecules were finally quantified via their GpL domain and normalized to the binding of the construct comprising the complete extracellular domain of TNFR2.

Supplemental Figure 3

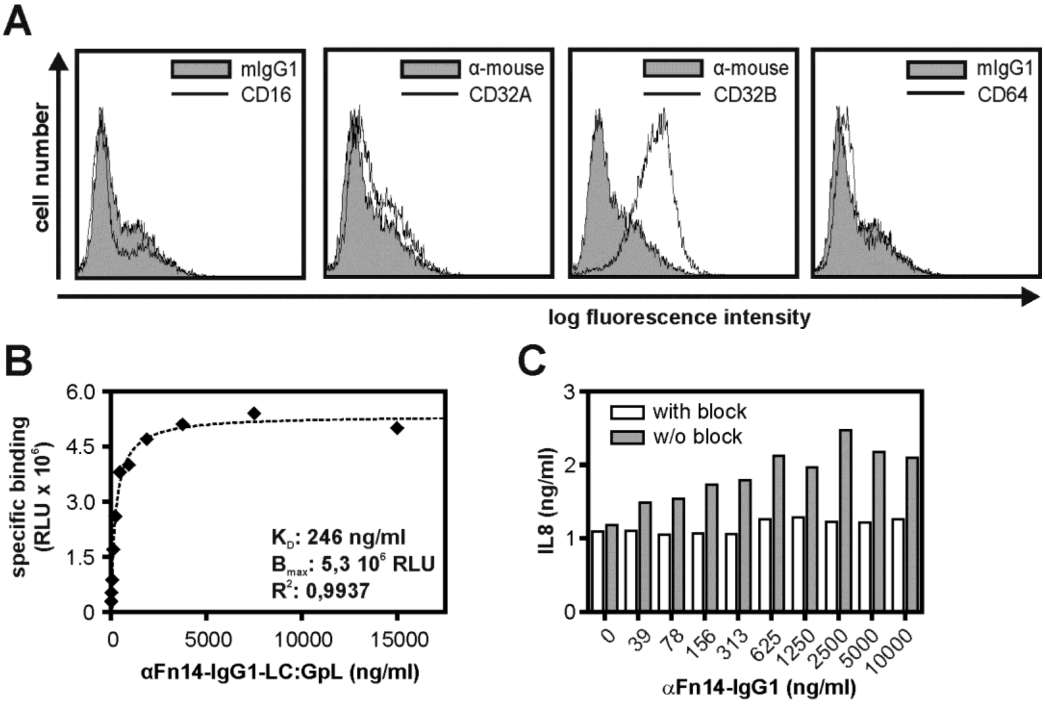

Fig. S3. (A) THP1 cells were analyzed for cell surface expression of the indicated FcγR by FACS. (B) Equilibrium binding of a GpL fusion protein of the IgG1 variant of the anti-Fn14 antibody 18D1 to THP1. (C) Cocultures of THP1 cells and Fn14-responsible WiDr cells were challenged with the indicated concentrations of anti-Fn14-IgG1 in the presence and absence of 100 µg/ml of an irrelevant IgG1 blocking the THP1-expressed FcγRs.

Supplemental Figure 4

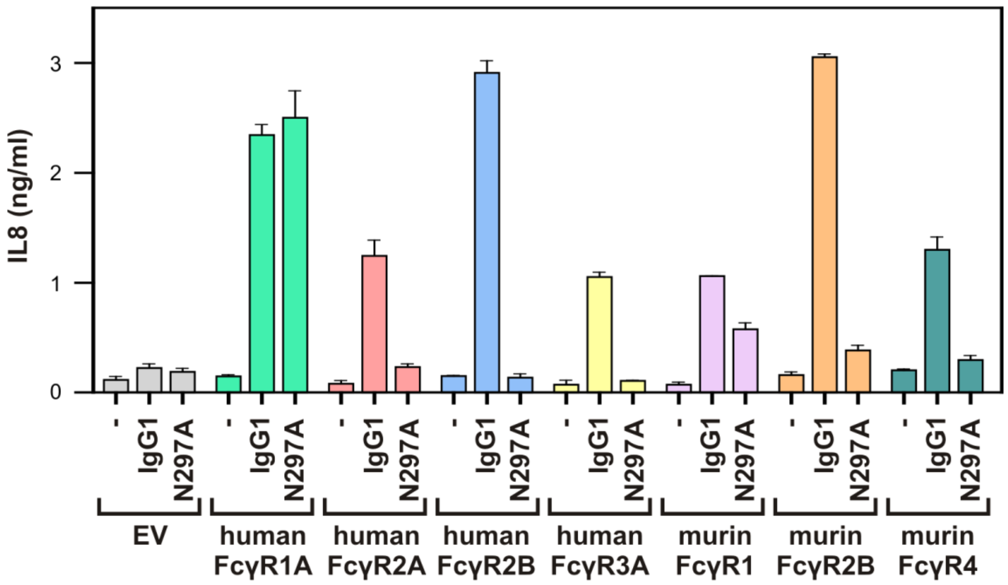

Fig. S4. Cocultures of HEK293 cells, transfected with empty vector (EV) or expression plasmids encoding the indicated FcγRs, and HeLa cells, which produce good amounts of IL8 after Fn14 stimulation, were treated with 1 µg/ml of anti-Fn14(18D1)-IgG1 or anti-Fn14(18D1)-IgG1(N297A). Next day, IL8 production was analyzed by ELISA to verify Fn14 activation.

Supplemental Figure 5

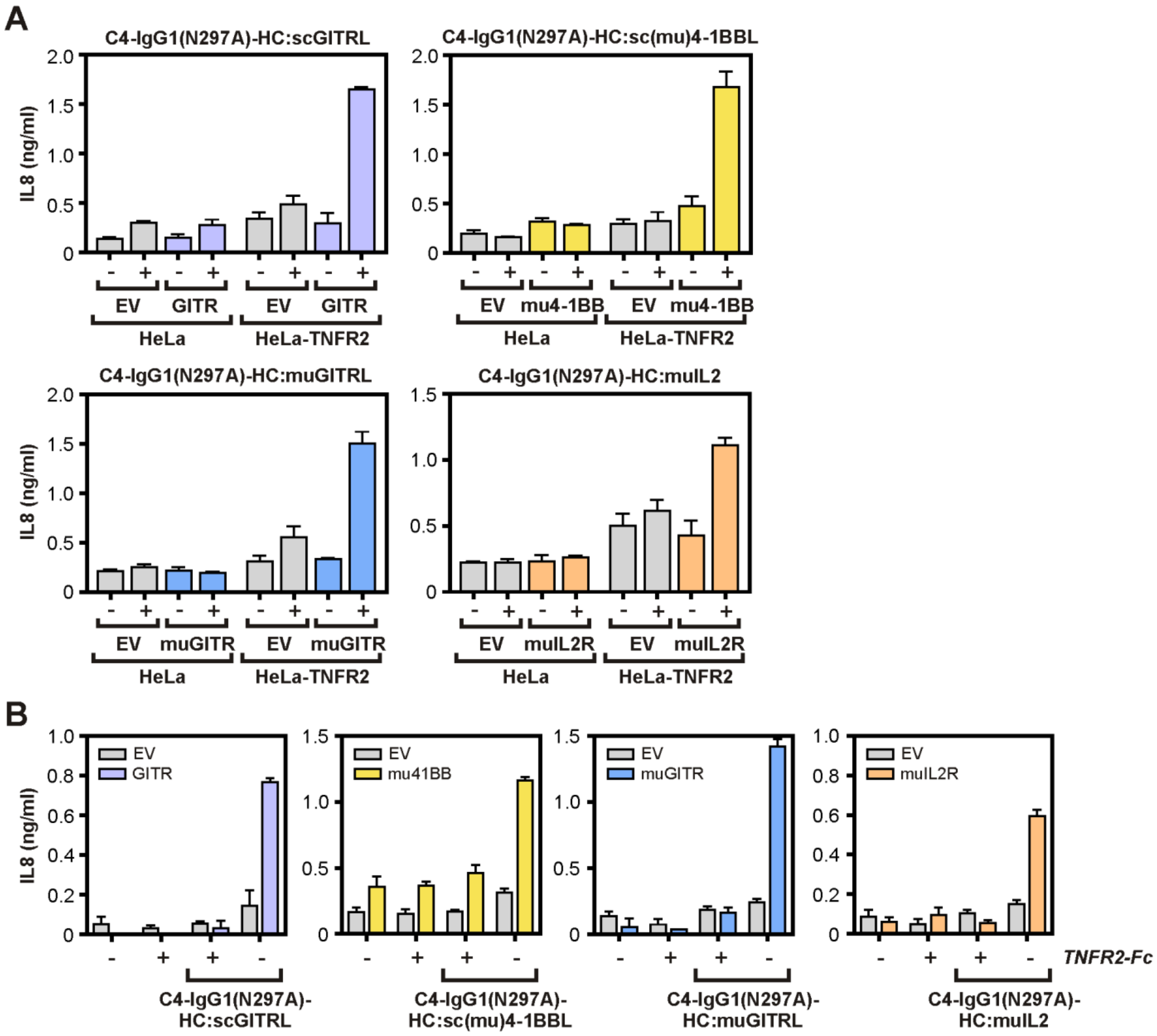

Fig. S5. (A) TNFR2-responsible HeLa-TNFR2 cells and as negative control conventional HeLa cells, which do not express TNFR2, were cocultivated with HEK293 cells which has been transfected with empty vector or plasmids encoding GITR, murine 4-1BB, murine GITR or the subunits of the murine IL2 receptor complex. Cocultures were challenged with 1 µg/ml of the indicated fusion proteins of the TNFR2-specific antibody C4 and next day IL8 production was determined. (B) Cocultures of HeLa-TNFR2 cells and HEK293 cells transfected as in “A” were stimulated with the various fusion proteins (1 µg/ml) in the presence and absence of 100 µg/ml TNFR2-Fc (Enbrel) and finally IL8 production was again determined to verify TNFR2 activation.

Supplemental Figure 6

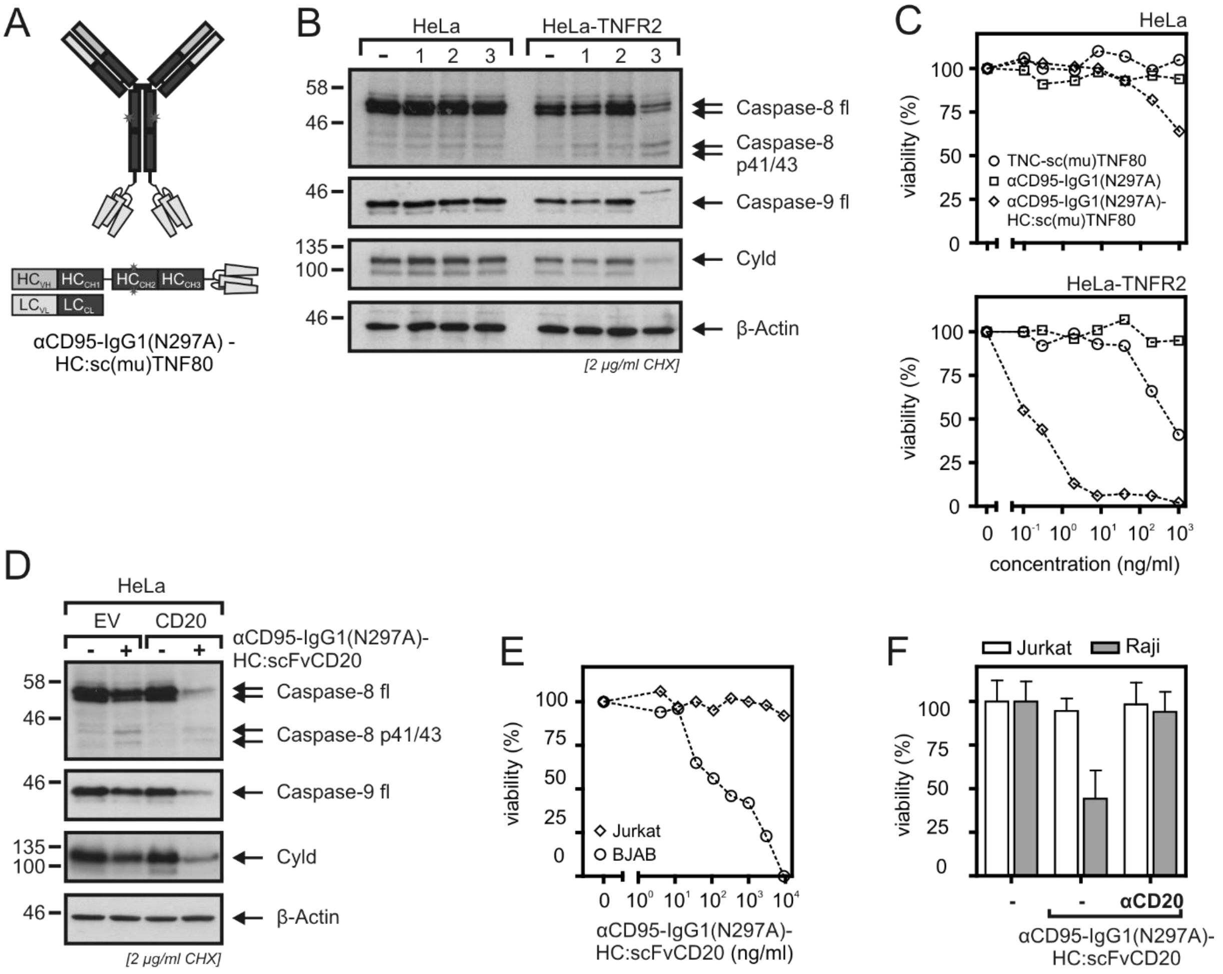

Fig. S6. An anti-CD95-IgG1 antibody gain high agonistic activity after cell surface target anchoring. (A) Domain architecture of anti-CD95-IgG1(N297A)-HC:sc(mu)TNF80. (B) HeLa cells, which do not express TNFR2, and TNFR2-responsible HeLa-TNFR2 cells were pretreated with 2 µg/ml CHX for 30 min and were then stimulated for 18 h with 400 ng/ml of the strong TNFR2 agonist TNC-sc(mu)TNF80 (1), with 3 µg/ml anti-CD95-IgG1(N297A) (2) or 3 µg/ml anti-CD95-IgG1(N297A)-HC:sc(mu)TNF80 (3). Finally, total lysates were analyzed with respect to the indicated proteins by western blotting. (C) Cells were pretreated with 2 µg/ml CHX for 30 min and were then stimulated for 18 h with the indicated concentrations of TNC-sc(mu)TNF80, anti-CD95-IgG1(N297A) or anti-CD95-IgG1(N297A)-HC:sc(mu)TNF80. Finally, cell viability was determined by crystal violet staining. (D) HeLa cells were cocultivated with HEK293 cells which have been transfected with empty vector or a CD20 expression plasmid. Cocultures were pretreated with 2 µg/ml CHX and challenged with 300 ng/ml anti-CD95-IgG1(N297A)-scFvCD20. Next day, expression of procaspases was evaluated by western blotting. (E) HeLa cells were pretreated with 2 µg/ml CHX and stimulated with the indicated concentrations of anti-CD95-IgG1(N297A)-scFvCD20 and CD20-negative Jurkat cells or CD20-positive BJAB cells. Next day, cellular viability was evaluated. (F) HeLa cells were cocultured with CD20-negative Jurkat cells and CD20-positive Raji cells. Cocultures were pretreated with 2 µg/ml CHX and, where indicated, with 50 µg/ml of the anti-CD20 mAb Rituximab. Cells were then challenged overnight with 100 ng/ml of anti-CD95-IgG1(N297A)-scFvCD20 and the following day cellular viability was determined by crystal violet staining.

Supplemental Figure 7

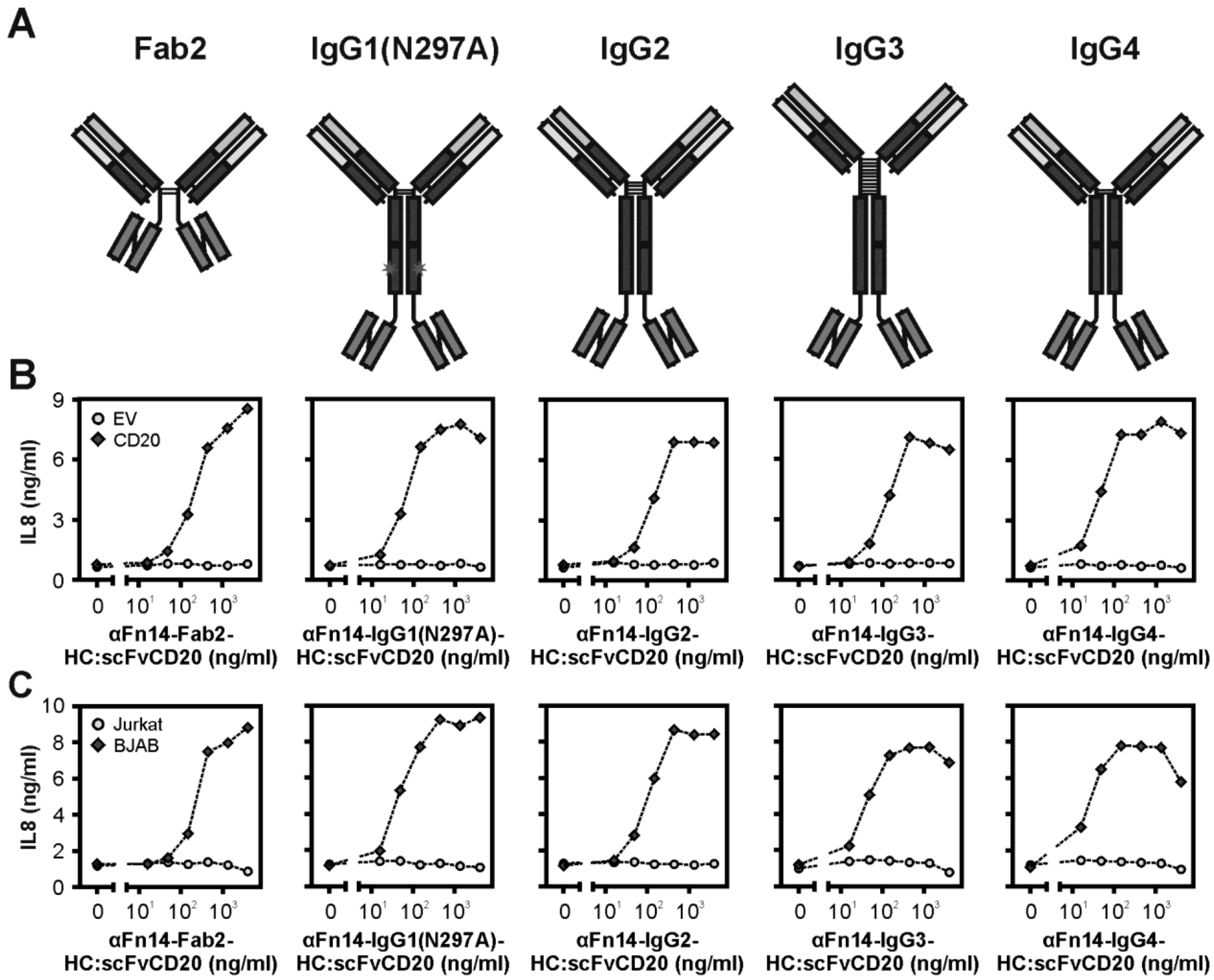

Fig. S7. Anti-Fn14-scFvCD20 isotype variants and anti-Fn14-Fab$_2$-HC:scFvCD20 activate Fn14 in CD20-restricted manner. (A) Domain architecture of the anti-Fn14 fusion proteins investigated. (B) WiDr cells were stimulated in triplicates with the indicated anti-Fn14 scFvCD20 fusion proteins along with HEK293 cells transfected with empty vector (EV) or a CD20-encoding expression plasmid. Next day, cell supernatants were analyzed for IL8 production. (C) WiDr cells were stimulated as in "B" in the presence of CD20-negative Jurkat cells instead of HEK293-EV transfectants and in the presence of CD20-positive BJAB cells instead of HEK293-CD20 transfectants. Again IL8 production was analyzed next day as an indicator of Fn14 activation.

Supplemental Figure 8

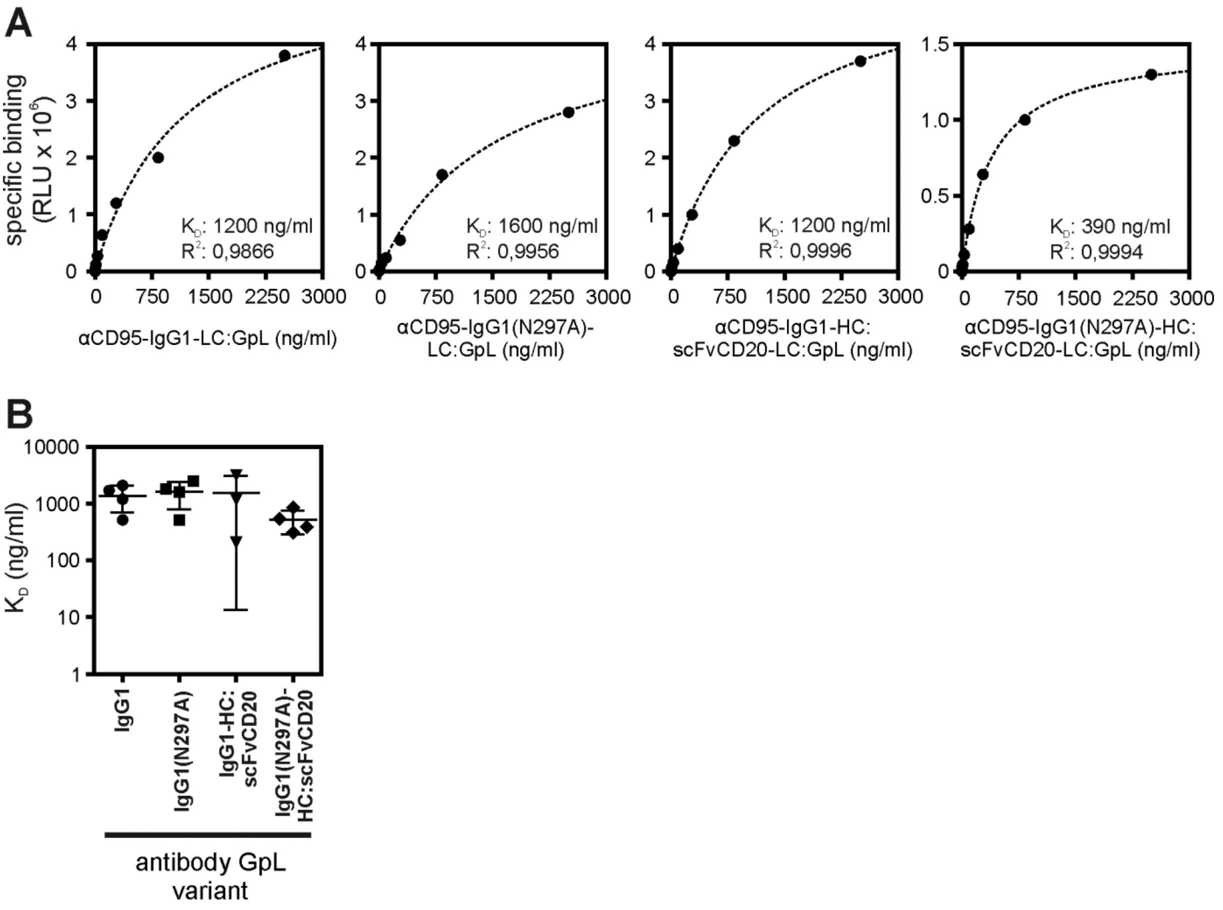

Fig. S8. Hek293 cells transiently expressing FcRn and empty vector transfected control Hek293 cells were aliquoted and incubated with increasing concentrations of indicated GpL antibody fusion proteins. After removal of the unbound molecules the remaining cell-associated luciferase activity was determined and specific binding values were calculated by subtracting the unspecific binding values obtained from the empty vector transfected cells from the total binding values obtained from the FcRn transfectants. Antibody fusion protein binding and the various washing steps have been performed at pH 5,5. The K_D-values were calculated with the "nonlinear regression to a one-site specific binding curve" function of the GraphPad Prism5 software. One representative binding experiment is shown for each of the various interactions in A. The diagram shown in B shows the KD-values of 3 or 4 independent experiments. All possible pairs of antibody variants have been analyzed according to Bonferroni's test but there was in no case a significant ($p < 0.05$) difference.
