# Supplementary material for: TNFRSF receptor-specific antibody fusion proteins with targeting controlled FcγR-independent agonistic activity
Source: Cell Death Dis. 2019 Mar 4;10(3):224. doi: 10.1038/s41419-019-1456-x (PMC6399339; doi:10.1038/s41419-019-1456-x)
Supplement: Supplementary file 2 — supplemental tables [file 41419_2019_1456_MOESM2_ESM.pdf]

Supplemental table I. TNFRSF receptor-specific antibodies evaluated in this study

Antigen	Isotype	Clone	Function	Applications	Company	Order-no.
**4-1BB**	mIgG1	4B4-1		FACS	BD Pharmingen ™	555956
**CD27**	mIgG1	57703	neutralization	FACS, WB	R&D Systems	MAB382
**CD40**	IgG1	G28.5 [a]				
**CD40**	mIgG1	HB14		FACS	Milteny Biotec	130-099-385
**CD40**	mIgG2A	LOB-11		FACS , IF, IHC, IP, WB	Santa Cruz	sc-13528
**CD95**	IgG1	E09 [a]		FACS		
**CD95**	mIgG1	DX2		IHC, WB	R&D Systems	MAB142
**Fn14**	hIgG1	18D1 [c]	blocking			
**Fn14**	mIgG2B	ITEM-4	neutralization	FACS, IF, WB	Invitrogen	12-9018-42
**Fn14**	hIgG1	P4A8 [d]	blocking			
**Fn14**	hIgG1	PDL192 [e]				
**GITR**	mIgG1	DT5D3		FACS	Milteny Biotec	130-099-088
**LTβR**	mIgG2B	31G4D8	agonist	ELISA , FACS, IP, WB,	Novusbio	NB100-78050
**LTβR**	mIgG1	71315	agonist	n.m.	R&D Systems	MAB6291
**LTβR**	mIgG1	71319		FACS , WB	R&D Systems	MAB629
**LTβR**	IgG1	BHA10 [f]				
**Ox40**	mIgG1	BER-Act35		FACS,IF, IHC, IF, WB	Santa Cruz	sc-20073
**TNFR1**	mIgG1	16803	neutralization	FACS, WB	R&D Systems	MAB225
**TNFR1**	mIgG1	MABTNFR1-A1		ELISA (capture)	BD Pharmingen ™	552535
**TNFR1**	mIgG2A	H398 [g]	blocking			
**TNFR1**	mIgG1	htr9	agonist, inhibits TNF-binding	FACS, IHC, WB	Acris	BM4053
**TNFR2**	mIgG2A	22235		FACS	R&D Systems	FAB226P
**TNFR2**	mIgG1	MR2-1		FACS , IHC, IP, WB	LSBio	LS-C139940
**TNFR2**	mIgG1	utr1	blocking	FACS , IHC, IP, WB	Acris	BM4054
**TNFR2**				ELISA (capture) [h]	R&D Systems	
**TRAILR1**	mIgG1	DJR1		FACS, IF	Biolegend ®	307202
**TRAILR1**	mIgG1	HS101	inhibition	FACS, ICC, IP	AdipoGen ®	AG-20B-0022
**TRAILR2**	mIgG1	D-6		ELISA , IF, IP, WB	Santa Cruz	sc-166624
**TRAILR2**	mIgG1	DJR2-2		FACS , IF	Biolegend ®	307302

[a] AJ853736 (PDB entry) [b] 3TJE_H+L (PDB entry) [c] sequence on request by Argenx

[d] Garber E, Burkly L, Michealson J, Lugovskoy A, Hsu YM, Hanf K. Anti-Fn14 antibodies and uses thereof. WO2009140177A2. 2009

[e] Culp P. Therapeutic use of anti-TWEAK antibodies. WO2009020933A2. 2009

[f] Michaelson et al., 2009 [1] [g] kind gift of Klaus Pfizenmaier [h] no additional information available

Supplemental table II. Primary structure of antibody chains used in this study

	CONSTRUCT	ARCHITECTURE
**[1]**	α4-1BB(HBBK4)-LC	L^{a} - F^{b} - V_{L} (US 2009/0041763 A1_H4B4-L) – C_{L}^{c}
**[2]**	α4-1BB(HBKK4)-IgG1(N297A)-HC-scFv:CD20	L^{a} - F^{b} – V_{H} (US 2009/0041763 A1_H4B4-H) – C_{H}^{d} – scFv:CD20^{f}
**[3]**	αCD40(G28.5)-LC	L^{a} - F^{b} - V_{L} (aa: 148-259 [AAB81500.1]) – C_{L}^{c}
**[4]**	αCD40(G28.5)-IgG1-HC	L^{a} - F^{b} – V_{H} (aa: 21-132 [CAH69220.1]) – C_{H}^{d}
**[5]**	αCD40(G28.5)-IgG1(N297A)-HC-scFv:CD20	L^{a} - F^{b} – V_{H} (aa: 21-132 [CAH69220.1]) – C_{H}^{e} – scFv:CD20^{f}
**[6]**	αCD95(E09)-LC	L^{a} - F^{b} - V_{L} (aa: 1-111 [3TJE_L]) – C_{L}^{c}
**[7]**	αCD95(E09)-IgG1-HC	L^{a} - F^{b} – V_{H} (aa: 1-132 [3TJE_H]) – C_{H}^{d}
**[8]**	αCD95(E09)-IgG1(N297A)-HC-scFv:CD20	L^{a} - F^{b} – V_{H} (aa: 1-132 [3TJE_H]) – C_{H}^{e} – scFv:CD20^{f}
**[9]**	αFn14(18D1)-LC	L^{a} - F^{b} - V_{L}^{h} – C_{L}^{c}
**[10]**	αFn14(18D1)-LC (murin)	L^{a} - F^{b} - V_{L}^{h} – C_{L} (aa: 3-111 [WP_077942483.1])
**[11]**	αFn14(18D1)-LC-GpL	L^{a} - F^{b} - V_{L}^{h} – C_{L}^{c} – GpL (aa: 63-230 [AEX33289.1])
**[12]**	αFn14(18D1)-IgG1-HC	L^{a} - F^{b} – V_{H}^{h} – C_{H}^{d}
**[13]**	αFn14(18D1)-IgG2-HC	L^{a} – V_{H}^{h} – C_{H} (aa: 137-464 [BAC85395.1])
**[14]**	αFn14(18D1)-IgG3-HC	L^{a} – V_{H}^{h} – C_{H} (aa: 141-519 [AAH89421.1])
**[15]**	αFn14(18D1)-IgG4-HC	L^{a} – V_{H}^{h} – C_{H} (aa: 118-446 [ASY91977.1])
**[16]**	αFn14(18D1)-mIgG1-HC	L^{a} – V_{H}^{h} – C_{H} (aa: 135-460 [BAQ25543.1])
**[18]**	αFn14(18D1)-mIgG2A-HC	L^{a} – V_{H}^{h} – C_{H} (aa: 136-467 [4WEB_H])
**[19]**	αFn14(18D1)-IgG1(N297A)-HC	L^{a} - F^{b} – V_{H}^{h} – C_{H}^{e}
**[20]**	αFn14(18D1)-Fab1-HC-scFv:CD20	L^{a} – V_{H}^{h} – C_{H} (aa: 117-232 [4OQT_H]) – scFv:CD20^{f}
**[21]**	αFn14(18D1)-IgG1(N297A)-HC-scFv:CD20	L^{a} - F^{b} – V_{H}^{h} – C_{H}^{e} – scFv:CD20^{f}
**[22]**	αFn14(18D1)-IgG2-HC-scFv:CD20	L^{a} – V_{H}^{h} – C_{H} (aa: 137-464 [BAC85395.1]) – scFv:CD20^{f}
**[23]**	αFn14(18D1)-IgG3-HC-scFv:CD20	L^{a} – V_{H}^{h} – C_{H} (aa: 141-519 [AAH89421.1]) – scFv:CD20^{f}
**[24]**	αFn14(18D1)-IgG4-HC-scFv:CD20	L^{a} – V_{H}^{h} – C_{H} (aa: 118-446 [ASY91977.1]) – scFv:CD20^{f}
**[26]**	αFn14(P4A8)-LC	L^{a} - F^{b} – V_{L} (aa: 311-421 [AIL25364.1]) – C_{L}^{c}
**[27]**	αFn14(P4A8)-IgG1-HC	L^{a} - F^{b} – V_{H} (aa: 437-557 [AIL25364.1]) – C_{H}^{d}
**[28]**	αFn14(PDL192)-LC	L^{a} - F^{b} – V_{L} (WO 2013/177386 Al_PDL192 V_{L}) – C_{L}^{c}
**[29]**	αFn14(PDL192)-IgG1-HC	L^{a} - F^{b} – V_{H} (WO 2013/177386 Al_PDL192 V_{H}) – C_{H}^{d}
**[30]**	αLTβR(BHA-10)-LC	L^{a} - F^{b} – V_{L} (aa: 1-107 [1]) – C_{L}^{c}
**[31]**	αLTβR(BHA-10)-IgG1-HC	L^{a} - F^{b} – V_{H} (aa: 1-113 [1]) – C_{H}^{d}
**[32]**	αTNFR2(C4)-LC	L^{a} - F^{b} - V_{L}^{i} – C_{L}^{c}

**[33]**	αTNFR2(C4)-LC (murin)	L^a - F^b - $V_L{}^i$ – C_L(aa: 2-111 [WP_077942483.1])
**[34]**	αTNFR2(C4)-IgG1-HC	L^a – $V_H{}^j$ – $C_H{}^d$
**[35]**	αTNFR2(C4)-IgG2-HC	L^a – $V_H{}^j$ – C_H (aa: 137-464 [BAC85395.1])
**[36]**	αTNFR2(C4)-IgG3-HC	L^a – $V_H{}^j$ – C_H (aa: 141-519 [AAH89421.1])
**[37]**	αTNFR2(C4)-IgG4-HC	L^a – $V_H{}^j$ – C_H (aa: 118-446 [ASY91977.1])
**[38]**	αTNFR2(C4)-mIgG1-HC	L^a – $V_H{}^j$ – C_H (aa: 137-460 [BAQ25543.1])
**[39]**	αTNFR2(C4)-mIgG2A-HC	L^a – $V_H{}^j$ – SSA – C_H (aa: 136-467 [4WEB_H])
**[40]**	αTNFR2(C4)-IgG1(N297A)-HC-muGITRL	L^a – $V_H{}^j$ – $C_H{}^e$ – muGITRL (aa: 50-177 [EDL39313.1])
**[41]**	αTNFR2(C4)-IgG1(N297A)-HC-muIL2	L^a – $V_H{}^j$ – $C_H{}^e$ – PGT – muIL2 (aa: 21-169 [AAA39281.1])
**[42]**	αTNFR2(C4)-IgG1(N297A)-HC-scFv:CD19	L^a – $V_H{}^j$ – $C_H{}^e$ – scFv:CD19[k] [2]
**[43]**	αTNFR2(C4)-IgG1(N297A)-HC-scFv:CD20	L^a – $V_H{}^j$ – $C_H{}^e$ – scFv:CD20[f]
**[44]**	αTNFR2(C4)-IgG1(N297A)-HC-scFv:CD70	L^a – $V_H{}^j$ – $C_H{}^e$ – V_H (US20090028872A1_2H5-VH) – LK[g] – V_L (US20090028872A1_2H5-VK)
**[45]**	αTNFR2(C4)-IgG1(N297A)-HC-scGITRL	L^a – $V_H{}^j$ – $C_H{}^e$ – GGGSG – GITRL[l] – LK[g] – GITRL[l] – LK[g] – QF – GITRL[l]
**[46]**	αTNFR2(C4)-IgG1(N297A)-HC-sc(mu)4-1BBL	L^a – $V_H{}^j$ – $C_H{}^e$ – GGGSG – mu4-1BBL[m] – LK[g] – mu4-1BBL[m] – LK[g] - mu4-1BBL[m]

[a] L = Leader: MNFGFSLIFLVLVLKGVQCEVKLVPR

[b] F = Flag: DYKDDDDK

[c] C_L = constant light (aa: 105-214 [BAA97671.1])

[d] C_H = constant heavy IgG1 (aa: 145-476 [AAA02914.1])

[e] IgG1(N297A): with A to N mutation at position 327 of constant heavy IgG1 [d]

[f] scFv:CD20 (aa: 38-280 [AUI40871.1]): V_H (Rituximab) – LK[g] – V_L (Rituximab)

[g] LK = Linker GGGGSGGGGSGGGGS

[h] sequence on request by Argenx

[i] V_L-C4: DIVMTQSHKFMSTSVGDRVSITCKASQDVDTAVAWYQQKPGQSPKLLIYWASTRHTGVPDRFTGSGSGTDYTLTISSVQAEDLARYYCQQYYSVPPTFGGGTKL

[j] V_H-C4: QVQLLQSGPELVKPGASVKLSCKASGYSFTSYDINWVKQRPGQGLEWVGWIYPRDGDTKYNEKFKGKAILTVDTSSNTAYMNLHSLTSEDSAVYFCARLTGPYWYFDVWGTGTTVTVSS

[k] kind gift of Prof. Helfrich (University of Groningen)

[l] GITRL (aa: 50-177 [AAD22634.1])

[m] mu4-1BBL (aa: 105-309 [NP_033430.1])

1. Michaelson JS, Demarest SJ, Miller B, Amatucci A, Snyder WB, Wu X, et al. Anti-tumor activity of stability-engineered IgG-like bispecific antibodies targeting TRAIL-R2 and LTbetaR. MAbs. 2009;1:128-41.

2. Stieglmaier J, Bremer E, Kellner C, Liebig TM, ten Cate B, Peipp M, et al. Selective induction of apoptosis in leukemic B-lymphoid cells by a CD19-specific TRAIL fusion protein. Cancer immunology, immunotherapy : CII. 2008;57:233-46.
